# Toxic but tasty – temporal dynamics and network architecture of heme‐responsive two‐component signaling in *Corynebacterium glutamicum*

**DOI:** 10.1111/mmi.14226

**Published:** 2019-03-22

**Authors:** Marc Keppel, Hannah Piepenbreier, Cornelia Gätgens, Georg Fritz, Julia Frunzke

**Affiliations:** ^1^ Institute of Bio‐ und Geosciences, IBG‐1: Biotechnology Forschungszentrum Jülich Jülich 52425 Germany; ^2^ LOEWE‐Zentrum für Synthetische Mikrobiologie Philipps‐Universität Marburg Marburg 35032 Germany

## Abstract

Heme is an essential cofactor and alternative iron source for almost all bacterial species but may cause severe toxicity upon elevated levels and consequently, regulatory mechanisms coordinating heme homeostasis represent an important fitness trait. A remarkable scenario is found in several corynebacterial species, e.g. *Corynebacterium glutamicum* and *Corynebacterium diphtheriae*, which dedicate two paralogous, heme‐responsive two‐component systems, HrrSA and ChrSA, to cope with the *Janus nature* of heme. Here, we combined experimental reporter profiling with a quantitative mathematical model to understand how this particular regulatory network architecture shapes the dynamic response to heme. Our data revealed an instantaneous activation of the detoxification response (*hrtBA*) upon stimulus perception and we found that kinase activity of both kinases contribute to this fast onset. Furthermore, instant deactivation of the P_*hrtBA*_ promoter is achieved by a strong ChrS phosphatase activity upon stimulus decline. While the activation of detoxification response is uncoupled from further factors, heme utilization is additionally governed by the global iron regulator DtxR integrating information on iron availability into the regulatory network. Altogether, our data provide comprehensive insights how TCS cross‐regulation and network hierarchy shape the temporal dynamics of detoxification (*hrtBA*) and utilization (*hmuO*) as part of a global homeostatic response to heme.

## Graphical abstract

Heme is an essential cofactor for almost all bacterial species but may cause severe toxicity upon elevated levels. In this study, we present a combined experimental and theoretical approach analyzing the temporal dynamics of heme detoxification and utilization in *Corynebacterium glutamicum,* which are orchestrated by the two two‐component systems ChrSA and HrrSA. These systems represent an interesting example of network evolution optimizing the cellular response to a multifaceted stimulus.

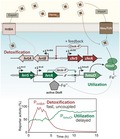

## Introduction

‘All things are poison, and nothing is without poison, the dosage alone makes it so a thing is not a poison’ – Paracelsus (1493–1541)

Heme represents an important iron source for almost all bacterial species (Andrews *et al.*, 2003) and is a ubiquitous cofactor of a variety of enzymes (Poulos, 2007). Elevated cellular concentrations of heme can, however, cause severe toxicity. But this is basically true for all nutrients as already emphasized by the Swiss physician and founder of modern toxicology, Paracelsus (Paracelsus, 1965; Borzelleca, 2000). Consequently, a robust regulation of homeostasis is key to the cell’s survival and typically, sophisticated regulatory mechanisms are engaged in maintaining optimal intracellular conditions and tolerance to environmental fluctuations.

Once inside the cell, most bacteria rely on heme oxygenases to catalyze the conversion of heme to biliverdin, thereby salvaging the central iron atom with the concomitant release of carbon monoxide (Wilks, 2002). One early‐characterized example for this class of enzymes is HmuO, a heme oxygenase of *Corynebacterium diphtheriae* that was found to be essential for the utilization of free and hemoglobin‐bound heme (Schmitt, 1997; Wilks and Schmitt, 1998). An ortholog of HmuO was also identified in *Corynebacterium glutamicum*, were the deletion of the corresponding gene led to reduced growth on hemin as sole iron source (Frunzke *et al.*, 2011). In Gram‐negative pathogens, including *Neisseria* spp. and *Pseudomonas aeruginosa*, proteins of the HemO/PigA family were found to catalyze the cleavage of the porphyrin ring structure, but do not share significant sequence similarity with HmuO of Gram‐positive species (Zhu *et al.*, 2000b; Ratliff *et al.*, 2001). The cost and benefit of using heme as an alternative iron source, however, needs to be carefully considered by the cell. Corynebacterial species, for example, employ the master regulator of iron homeostasis, DtxR, to feed information on iron availability into the network controlling heme homeostasis. DtxR was shown to repress *hmuO* under iron‐replete conditions and thereby adds an additional layer of regulation to the physiological response to heme (Schmitt, 1997; Wennerhold and Bott, 2006).

Due to the reactive nature of the heme molecule, high levels are readily toxic to microbial cells (Imlay *et al.*, 1988; Nir *et al.*, 1991; Anzaldi and Skaar, 2010; Wakeman *et al.*, 2012). Consequently, organisms have evolved a variety of mechanisms to minimize toxic effects. Whereas some bacteria rely mostly on their oxygenase to degrade excess heme, such as *Neisseria gonorrhoeae *(Zhu *et al.*, 2000a) or *Clostridium perfringens* (Hassan *et al.*, 2010), an alternative strategy can be found in the eukaryotic parasite *Plasmodium s*pp. which is capable to sequester excess heme in an insoluble substance called hemozoin (Fitch, 1998; Jani *et al.*, 2008; Anzaldi and Skaar, 2010). While several bacterial species harbor bacterioferritins, which can store iron in the form of heme molecules, other forms of sequestration are not well described so far (Andrews *et al.*, 2003; Anzaldi and Skaar, 2010). A third class of detoxification systems are heme exporters, such as HrtBA which have been described for several Gram‐positive species including *Staphylococcus aureus* (Torres *et al.*, 2007; Stauff and Skaar, 2009a), *Bacillus anthracis* (Stauff and Skaar, 2009a), *Streptococcus agalactiae* (Fernandez *et al.*, 2010) and can also be found in corynebacterial species (Bibb and Schmitt, 2010; Heyer *et al.*, 2012).

Bacterial two‐component systems (TCS), consisting of a membrane bound histidine kinase (HK) and a cytoplasmatic response regulator (RR) (Mascher *et al.*, 2006; Zschiedrich *et al.*, 2016), play a central role as transient heme sensor systems in Gram‐positive species (Stauff and Skaar, 2009b). This is known from bacteria such as *Staphylococcus aureus* and *Bacillus anthracis*, both utilizing the heme sensor system HssRS to react to heme as extracellular stimulus (Stauff and Skaar, 2009a; 2009b). A common theme among *Corynebacteriaceae* appears to be the dedication of two paralogous TCS for the regulation of heme homeostasis (Bibb *et al.*, 2007; Frunzke *et al.*, 2011; Bott and Brocker, 2012; Heyer *et al.*, 2012; Burgos and Schmitt, 2016). Here, the HrrSA and ChrSA systems coordinate the expression of genes involved in heme biosynthesis, heme detoxification (*hrtBA*), respiratory chain and the heme oxygenase (*hmuO*). While in *C. glutamicum* it was suggested that both TCS have partially overlapping regulons, HrrSA was shown to play an important role in in the utilization of heme as an alternative iron source by activating expression of *hmuO*, whereas ChrSA is crucial for the activation of the *hrtBA* operon encoding a heme exporter (Frunzke *et al.*, 2011; Heyer *et al.*, 2012). Furthermore, previous studies of our group revealed significant cross‐phosphorylation between these TCS but a highly specific phosphatase activity of the HKs toward their cognate RR (Hentschel *et al.*, 2014). Previous studies focused mostly on the identification of target genes, which were confirmed by different *in vivo* and *in vitro* assays. However, the systemic understanding of this homeostatic network, maintaining balance between heme detoxification and utilization, demands the analysis of temporal dynamics and requires comprehensive insights in the particular network architecture.

In this study, we have conducted an analysis of reporter assays of HrrSA and ChrSA target promoters in the background of the wild‐type strain, as well as in mutant strains lacking single components of the two TCSs. These data were integrated in a quantitative mathematical model, which was used to test functional hypotheses and to simulate distinct differences in autoregulation and ON/OFF kinetics of target promoters. Finally, by studying the impact of the iron regulator DtxR on *hrrA *and *hmuO *expression at temporal resolution our data as well as the model revealed that DtxR adds an important additional regulatory level ensuring the appropriate timing of heme utilization.

## Results

### Temporal hierarchy in the heme utilization and detoxification response

**Figure 1:**
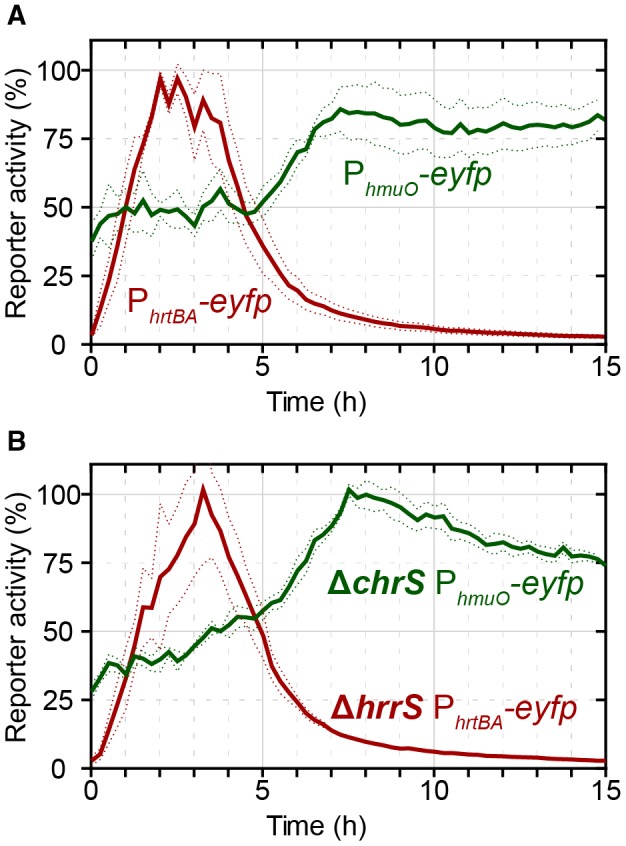
Activation of P_*hmuO*_ and P_*hrtBA*_ in response to extracellular heme addition. A. The *C. glutamicum* wild‐type strain was transformed with one of the target gene reporters pJC1_P_*hmuO*_‐*eyfp* or pJC1_P_*hrtBA*_‐*eyfp*. Iron‐deprived cells were subsequently cultivated in a microbioreactor system (Biolector) in CGXII minimal medium with 2% (w/v) glucose containing 4 µM hemin. The eYFP fluorescence was measured as the output of target promoter activation, and backscatter values were recorded to monitor biomass formation. The specific fluorescence (fluorescence/backscatter) was normalized according to material and methods and the reporter activity (%) was calculated with the maximum reporter output. B. *C. glutamicum* Δ*chrS*/pJC1_P_*hmuO*_‐*eyfp* and Δ*hrrS*/pJC1_P_*hrtBA*_‐*eyfp* grown as described in (A). Non‐cognate sensor kinases do not significantly affect the response profile. [Colour figure can be viewed at wileyonlinelibrary.com]

Under iron‐limiting conditions, the growth of *C. glutamicum* is significantly impaired, but can be restored by the presence of heme in the medium. Provided that excess heme is toxic to the cells, we wondered which strategy *C. glutamicum* uses to regulate the balance between its heme utilization and detoxification modules. To this end, we studied the expression dynamics of the two major components responsible for heme utilization (*hmuO*) and detoxification (*hrtBA*) in response to an extracellular heme stimulus, by monitoring promoter‐reporter fusions for the two systems (Hentschel *et al.*, 2014). Interestingly, a wild‐type strain of *C. glutamicum* transformed with plasmids carrying the reporter constructs (pJC1_P_*hmuO*_ *‐eyfp *or pJC1_P_*hrtBA*_ *‐eyfp*) revealed highly distinct response profiles and a temporal hierarchy in reporter output of the P_*hmuO*_ and P_*hrtBA*_ promoters (Fig. 1): While we observed a nearly instant but transient response for the heme detoxification module *hrtBA* to 4 µM extracellular heme (Fig. 1, *red line*), the heme utilization module *hmuO* displayed higher initial expression levels compared to *hrtBA* and experienced an expression boost after a delay of about 5 h (Fig. 1, *green line*). This increase in *hmuO* expression temporally coincides with a declining* hrtBA *expression. From a physiological perspective, these antagonistic expression profiles seem plausible and impressively demonstrate the urgency of detoxification over utilization after first contact with the stimulus.

How does *C. glutamicum* implement appropriate timing of detoxification and utilization using two paralogous TCS responsive to the same stimulus? In this study, we formulated three distinct questions and addressed those by experiments described in the following:

1. How does cross‐regulation between ChrSA and HrrSA affect *hrtBA* and *hmuO* expression respectively?
2. Does the differential interpretation of their common stimulus, i.e. the external heme concentration, impact the response?
3. How does regulatory hierarchy and network architecture affect the response profile?

To test the first hypothesis, we tested two mutant strains deleted for either one of the HKs (Δ*chrS *and Δ*hrrS*) and transformed them with a reporter plasmid carrying the non‐cognate target promoter (Δ*chrS/*pJC1_P_*hmuO*_‐*eyfp* and Δ*hrrS/*pJC1_P_*hrtBA*_‐*eyfp*). Strikingly, despite some quantitative differences (as discussed below), neither of the deletions changed the qualitative response of the non‐cognate target promoter toward heme (Fig. 1B), that is the P_*hrtBA*_‐*eyfp* response was still transient in a Δ*hrrS* mutant and the P_*hmuO*_‐*eyfp* response was still delayed in a Δ*chrS* mutant, indicating that cross‐regulation between the TCS cannot explain the antagonistic regulation strategy in *C. glutamicum*.

### Modeling of heme uptake and consumption

**Figure 2:**
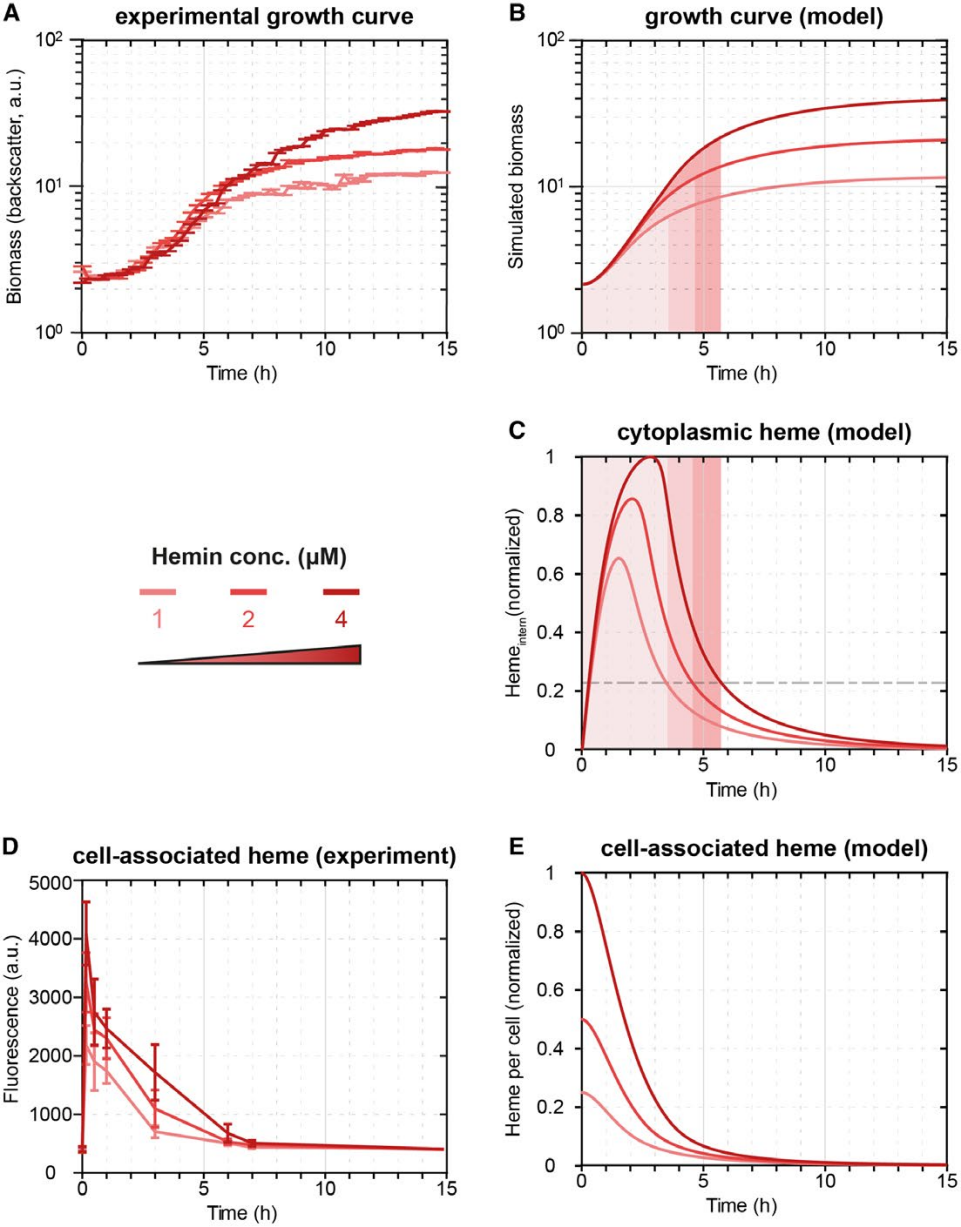
The mathematical model reproduces the experimental growth curves quantitatively. A. Growth curves of the *C. glutamicum* wild‐type strain under increasing hemin concentrations (1 μM, 2 μM and 4 μM). B. The mathematical model can reproduce the average growth behavior (left). C. The mathematical model predicts the consumption of cytoplasmic heme required for growth. D. The depletion of cell‐associated heme (sum of internal heme and external heme adhering to the cell) dictates the bacterial growth. The experimental data align with (E) the model predictions of total heme levels per cell (cytoplasmic heme and the portion of external heme per cell). [Colour figure can be viewed at wileyonlinelibrary.com]

Therefore, we wanted to test whether the depletion of external heme could serve as a joint trigger to cause opposing regulation of heme utilization and detoxification systems. However, before turning to this question, we first asked how long it would take to deplete heme in our experiments? To this end, we considered a simple mathematical model describing the uptake and consumption of heme, assuming that the reproduction of *C. glutamicum *requires ~5 × 10^6^ Fe^2+^ molecules per single cell (see Supplementary Text for details). Since this number sets a constraint on the growth kinetics of *C. glutamicum* in the heme‐supplied medium, we studied the availability of heme per cell during bacterial growth. At the given final biomass and at the experimentally determined growth rate in our medium (Fig. 2A and B), the model predicts a depletion of the total heme levels per cell (levels of cytoplasmic heme and portion of extracytoplasmic heme in the medium per cell) approx. 3–5 h after the start of the experiment (Fig. 2E), depending on the initial heme concentrations in the medium. Experimental measurements of the levels of cell‐associated heme (see Experimental procedures for experimental details) (Fig. 2D), which correspond to the model predictions of the heme levels per cell, confirm these dynamics and point out that the availability of heme in the medium dictates the growth dynamics of *C. glutamicum*. Hence, when comparing the experimental growth curves (Fig. 2A) with the theoretical predictions, the time points when cytoplasmic heme pools are depleted (Fig. 2C), in fact correlate with the cease of growth of the cultures in experiment and theory (Fig. 2A and B). Also the time point of growth cessation can be tuned by adding different initial heme concentrations (1–4 μM) to the medium (Fig. 2A), as predicted by our model (Fig. 2B).

### Transient expression of the *C. glutamicum hrtBA* detoxification module

**Figure 3:**
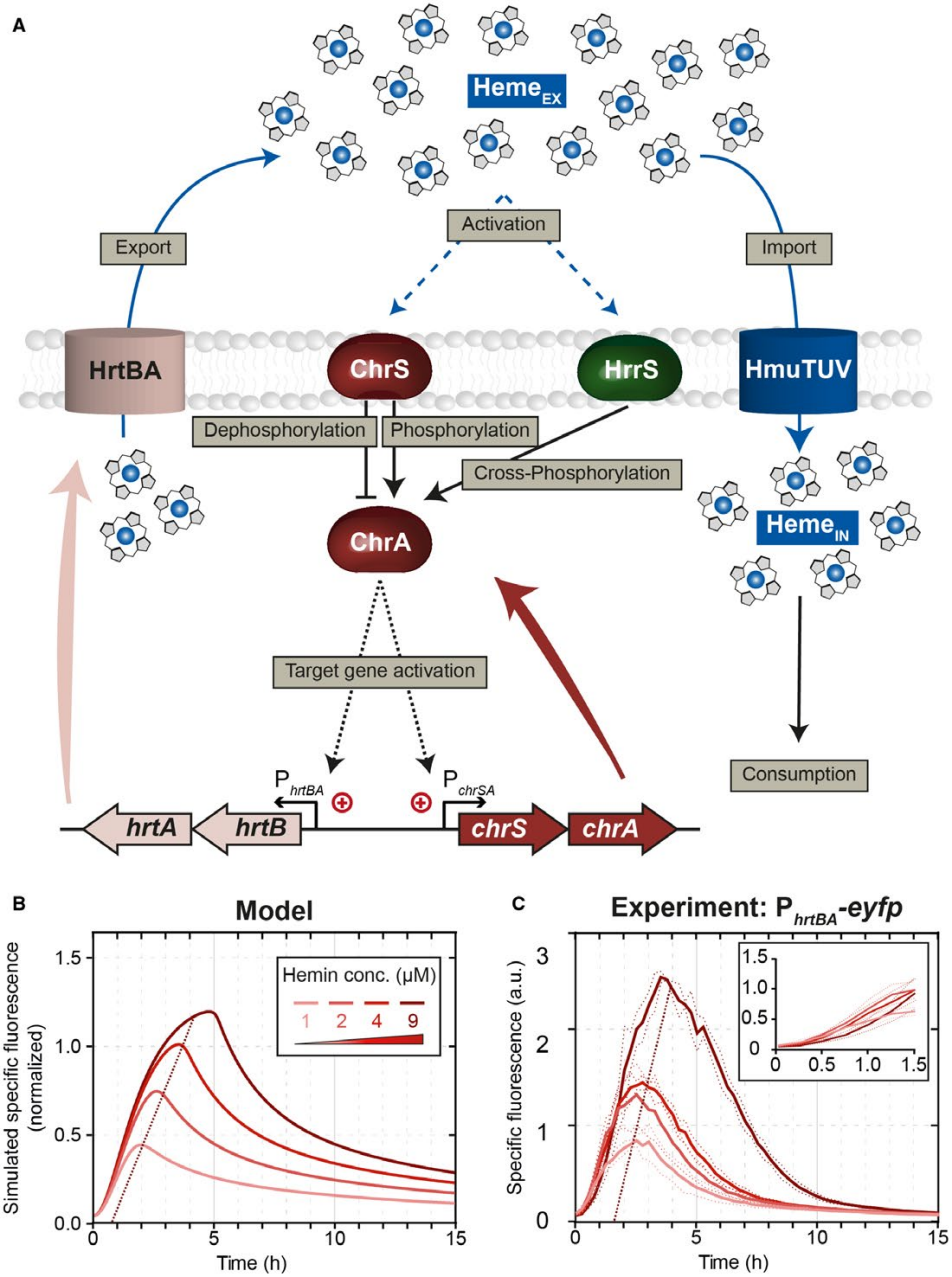
Regulatory scheme and dynamic response of the *C. glutamicum* heme detoxification module. A. Scheme of the regulatory interactions considered in the mathematical model for the heme detoxification module. Uptake of external heme molecules via HmuTUV and subsequent consumption/incorporation via diverse enzymes is crucial for bacterial growth under iron starvation. The fine‐tuned response to heme in order to avoid intoxication is mainly based on the two TCSs ChrSA and HrrSA. The two kinases ChrS and HrrS are autophosphorylated in response to external hemin. After activation, they (cross‐)phosphorylate the response regulator ChrA. In addition, the non‐phosphorylated form of ChrS functions as a phosphatase on the phosphorylated response regulator ChrA. The phosphorylated response regulator activates expression of its target genes *hrtBA* and *chrSA*. The gene product of *hrtBA* is a heme exporter that transports internal heme to the extracellular space. B. Dynamical response of our computational model for the detoxification module (Supplementary Text; Model equations M1) toward different external heme levels, as given by the simulation of specific fluorescence of a P_*hrtBA*_‐*eyfp* reporter, normalized to the maximal specific fluorescence at 4 μM heme. C. Experimental dynamical response of a P_*hrtBA*_‐*eyfp* reporter in wild‐type *C. glutamicum* cells toward different heme concentrations supplied in the medium at *t* = 0 h. [Colour figure can be viewed at wileyonlinelibrary.com]

Next, we asked whether the depletion of heme could also explain the transient activity of the *hrtBA* promoter. To this end, we extended our mathematical model to describe stimulus perception and regulation within the two TCSs, as well as the dynamical response of the *chrSA* and *hrtBA* operons (for details, the reader is referred to the Supplementary text). Briefly, the model considers sensing of externally added heme and subsequent autophosphorylation of ChrS and HrrS (Keppel *et al.*, 2018), (cross‐)phosphorylation of ChrA by phosphorylated HKs (ChrS~P and HrrS~P) and promoter activation of P_*chrSA*_ and P_*htrBA*_ by phosphorylated response regulator ChrA~P (Fig. 3A). Simulations of the model predict that the depletion of external heme dictates the time point of deactivation of P_*hrtBA*_, leading to a prolonged promoter activity and higher overall HrtBA production at higher initial heme concentrations (Fig. 3B). In fact, when experimentally supplying different heme concentrations (1–9 μM) to the medium, we found that both the strength as well as the duration of P_*hrtBA*_‐*eyfp* expression increased with increasing amounts of supplied heme (Fig. 3C). In the light of a bifunctional ChrS kinase/phosphatase, exhaustion of external heme is sufficient to explain the transient response dynamics of P_*hrtBA*_‐*eyfp* expression.

**Figure 4:**
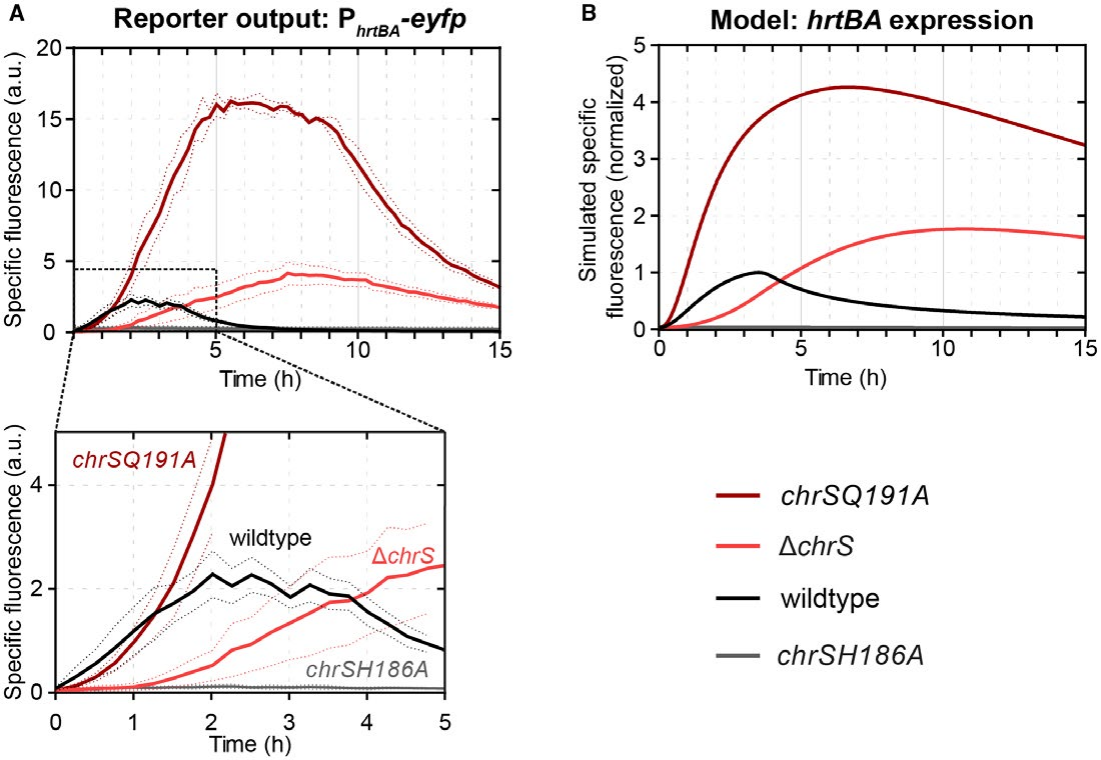
Kinase and phosphatase activity of ChrS shape the* hrtBA* response. A. Reporter output of *C. glutamicum* wild type and mutant strains carrying the vector pJC1_P_*hrtBA*_‐*eyfp*. Cells were inoculated in CGXII minimal medium with 2% (w/v) glucose containing 4 µM hemin as iron source. B. Simulated specific fluorescence of the *C. glutamicum* wild‐type strain and in the mutant strains *chrSQ191A* (phosphatise = off), *chrSH186A* (kinase = off) and Δ*chrS*. [Colour figure can be viewed at wileyonlinelibrary.com]

Interestingly, our experimental data also showed that for all heme concentrations, the initial response (within the first hour) of the reporter P_*hrtBA*_‐*eyfp* was almost identical (Fig. 3C) and that the overall peak height is only modulated by the time point of stimulus decline. This suggests that either (i) despite varying levels of ChrA~P, the P_*hrtBA*_ promoter is nearly fully saturated or (ii) the applied heme concentrations lead to saturation of the sensor kinase, i.e. maximal phosphorylation ([ChrS~P]/[ChrS_TOT_] ≈1), and thus to similar phosphorylation levels of ChrA. In order to discriminate between these scenarios, we sought to increase ChrA~P levels in the cell and test whether the P_*hrtBA*_ will be more active than in the wild type. To this end, we analyzed a *chrS* phosphatase‐OFF mutant (*chrSQ191A*) still harboring its kinase activity (Hentschel *et al.*, 2014), supposedly leading to higher ChrA~P levels. Interestingly, the *chrSQ191A* phosphatase mutant strain displayed a sevenfold increased P_*hrtBA*_‐*eyfp *output (Fig. 4A), as incurred by a higher and more sustained promoter activity within the first 4 h of incubation when compared to the wild type (Fig. 4A *inset*). A similar behavior can also be observed in our computational models, in which the maximal phosphorylation level of ChrA is 25% in the wild type as compared to 100% in the phosphatase mutant (Fig. S5B), leading in our model to a stronger initial promoter activity in the latter case (Figs. 4B and S5C). The predicted increase of initial promoter activity is, however, more prominent in the model than in our experimental data, in which the kinase mutant showed a wild‐type‐like behavior for around 1 h before reaching a stronger promoter activity (Fig. 4A *inset*). Within the model, the time point of differentiation between the reporter output of the wild type and the phosphatase‐OFF mutant – induced by stimulus decline and accordingly divergent ChrA~P levels (Fig. S5B) – is reached after ≈ 15 min (Fig. S5C). This discrepancy emphasized that saturation of kinase activity is not sufficient to explain the experimental data and that instantaneous promoter occupancy by phosphorylated response regulator may contribute to the fast onset of the detoxification response (see ‘Memoryless activation of the *hrtBA* detoxification module’).

Finally, another interesting feature of the model is that the depletion of external heme leads to a quick dephosphorylation of ChrA~P and promoter shut‐off in the wild type. In contrast, in the phosphatase mutant, ChrA~P is only slowly diluted and/or degraded by growth or spontaneous dephosphorylation (Fig. S5B), leading to a significantly delayed promoter shut‐off about 1 h after external heme depletion. Thus, the strong phosphatase activity of ChrS is important in wild type cells in order to quickly turn off *hrtBA* expression once external heme is depleted.

### Both kinases, ChrS and HrrS, contribute to a fast onset of the P_*hrtBA*_ promoter

Next, we wanted to study the response of the heme detoxification module in a strain featuring reduced ChrA~P levels. However, a *chrS* mutant deficient in its kinase activity (*chrSH186A*) was unable to activate the P_*hrtBA*_ promoter altogether (Fig. 4A; *grey line*), suggesting that ChrSH186A retains its strong phosphatase activity and likely reduces ChrA~P below a level required to activate P_*hrtBA*_. Instead, our model predicted that in a Δ*chrS* mutant, which lacks both kinase and phosphatase activity from ChrS, the non‐cognate HrrS sensor kinase should be able to slowly, but gradually phosphorylate ChrA~P (Hentschel *et al.*, 2014) and thus activate P_*hrtBA*_ (Fig. 4B; *light red*). Indeed, the experimental kinetics of the P_*hrtBA*_ ‐*eyfp* reporter showed a weaker activation during the first 2 h, but also displayed a more sustained and eventually a stronger expression peak compared to the wild type (Fig. 4A). Within our computational model, this sustained response is again caused by the slow rate of dilution and/or dephosphorylation of ChrA~P after heme depletion, given the lack of phosphatase activity in the Δ*chrS* mutant (Fig. 4B). Taken together, these data show that in the absence of ChrS, the non‐cognate kinase HrrS is sufficient to activate the promoter of the detoxification module, P_*hrtBA*_.

**Figure 5:**
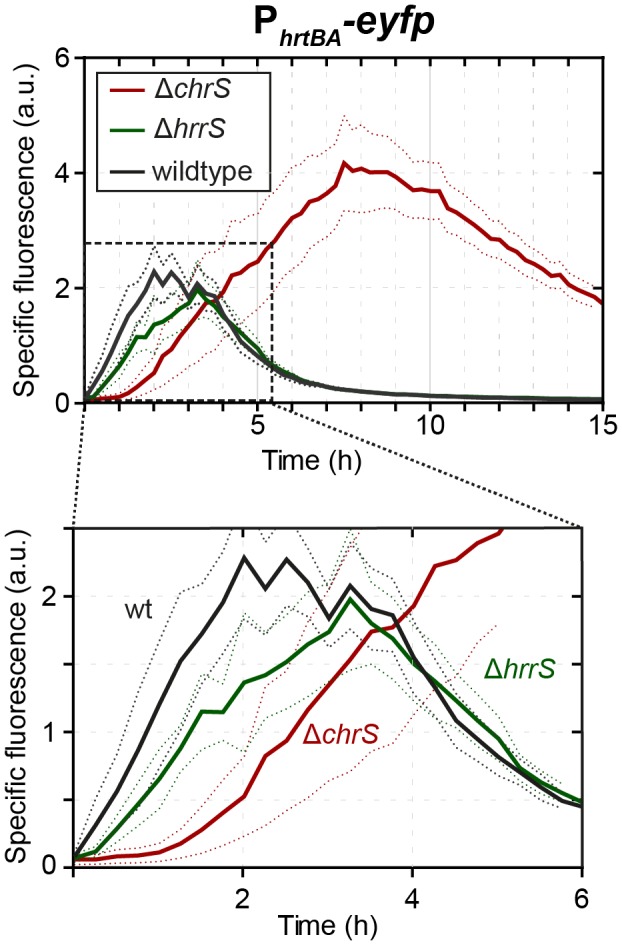
HrrS‐mediated cross‐phosphorylation of ChrA might act as a kick‐start impulse contributing to a fast ON‐set of the P_*hrtBA*_ response. Reporter output of *C. glutamicum* wild‐type cells and the mutant strains ∆*hrrS* and ∆*chrS*, carrying the vector pJC1_P_*hrtBA*_‐*eyfp* and cultivated in CGXII minimal medium with 2% (w/v) glucose containing 4 µM hemin as iron source. [Colour figure can be viewed at wileyonlinelibrary.com]

This provoked the question as to whether the non‐cognate kinase HrrS also has an effect on the induction kinetics of P_*hrtBA*_ in wild‐type cells. Interestingly, when measuring the P_*hrtBA*_‐*eyfp* reporter activity in a Δ*hrrS* mutant, the mutant indeed showed a delayed promoter activation and was about 20–30 min slower than the wild type (Fig. 5). This suggests that HrrS acts as a ‘kick‐starter’ in order to speed up the induction of the detoxification system. The additional kinase activity conferred by HrrS might in fact be needed as a support for ChrS to achieve higher ChrA phosphorylation levels, given that our analysis above suggested that ChrS is already fully in its kinase state for all heme concentrations applied here.

### Memoryless activation of the *hrtBA* detoxification module

Above‐described results suggested that a high promoter occupancy by ChrA~P may contribute to the fast onset of the detoxification response. Considering this scenario, we would not expect memory in the P_*hrtBA*_ response if two heme pulses were applied at subsequent times. Theoretically, our model predicted nearly identical levels of promoter saturation for different heme levels, suggesting that the application of a second heme pulse should not lead to a faster response and no priming effect on the output should be observable.

**Figure 6:**
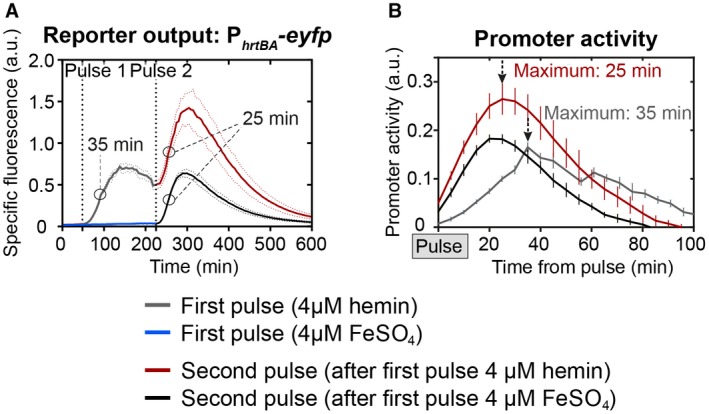
Additional heme pulses do not ‘prime’ the P_*hrtBA*_ output. A. *C. glutamicum* wild type cells were transformed with the target gene reporter pJC1_P_*hrtBA*_‐*eyfp* and starved from iron overnight as described in material and methods. Subsequently, the cells were inoculated in CGXII minimal medium without iron source and eYFP fluorescence (=reporter output) and backscatter (biomass) was measured every 5 min. After 45 min, hemin (A, grey line) or FeSO_4_ (A, black line) was added to a final concentration of 4 µM. A second pulse of 4 µM hemin was applied to both conditions after 225 min. B. Promoter activities (calculated as derivative of the fluorescence intensity (I) over time (t), divided by the cell’s biomass (backscatter), that is $\frac{dI\left(t\right)}{\mathrm{dt}}/backscatter\left(t\right)$) of (A) in relation to the first or second pulse. [Colour figure can be viewed at wileyonlinelibrary.com]

While lag phase cells (growth depicted in Fig. S1) needed up to 35 min to reach maximum promoter activity following a heme pulse, promoter activity was nearly instantaneously observable after an additional heme pulse in the early exponential phase (Fig. 6A). Here, cells did respond equally fast irrespective of whether the cells were primed first, but a slightly higher maximal activity was observed after cells were primed with a heme pulse (Fig. 6A, *red line*) in comparison to an iron pulse (Fig. 6A, *black line*). Furthermore, the time point of the second heme pulses did not affect the onset of the P_*hrtBA*_ response (Fig. S2). Please note that the higher amplitude of the second pulse (Fig. 6A) is a result of cells already containing eYFP molecules and a ‘reactivation’ of ChrSA (and thus P_*hrtBA*_) leads to formation of additional eYFP molecules. The total increase is comparable to the increase in the first 4 µM hemin pulse. Overall, this data supports the hypothesis that promoter saturation by phosphorylated response regulator contributes as a significant determinant of response kinetics.

### Heme utilization is co‐regulated by DtxR integrating information on iron availability

While a detoxification response must be fast and rather uncoupled from other regulatory interference, utilization of a particular nutrient has to be carefully considered in the light of the current physiological status of the cell: As shown above, the activation of the detoxification module *hrtBA* is solely influenced by the amount of heme in the medium. In contrast, for a decision on heme utilization as an alternative iron source, information on general iron availability needs to be incorporated into the network controlling* hmuO* expression. In this context, it was already revealed by the previous studies that both *hrrA* and *hmuO* are repressed by the iron regulator DtxR in response to iron availability (Wennerhold and Bott, 2006).

**Figure 7:**
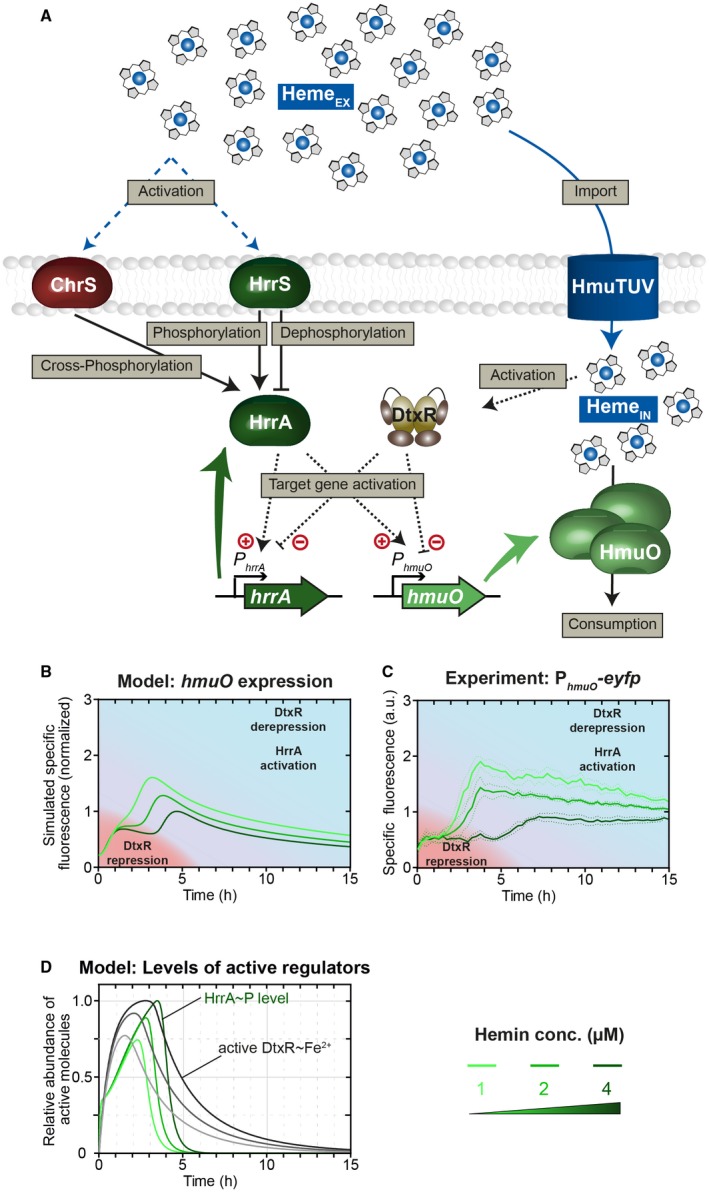
Information on iron availability is integrated into the HrrSA‐regulated utilization module *hmuO* via DtxR. A. The mathematical model of the heme utilization module in *C. glutamicum* shares many basic assumptions with the model of heme detoxification (for description see Fig. 3). Here, heme consumption is assumed to be supported by the heme oxygenase HmuO, whose production is regulated by the phosphorylated response regulator HrrA~P and the iron repressor DtxR. The activation of DtxR is expected to be influenced by internal heme levels. DtxR repression and HrrA activation shape the delayed response of the heme utilization module *hmuO*. B. The P_*hmuO*_ promoter is activated by the phosphorylated response regulator HrrA~P after a significant time delay of 3–5 h. Higher heme concentrations lead to a prolonged delay and a lower *hmuO* expression in general. C. The mathematical model of the heme utilization network in *C. glutamicum* could reproduce this behavior and gave an explanation regarding. D. The temporal dynamics of both regulators on P_*hmuO*_. Levels of the activated iron repressor DtxR increase immediately after addition of heme and repress the promotor activation of P_*hmuO*_, proportional to stimulus strength. However, HrrA~P levels increase with a short time delay in response to the stimulus and activate the promotor to a certain extend at the beginning and with increasing intensity upon DtxR dissociation [Colour figure can be viewed at wileyonlinelibrary.com]

Given that DtxR repression and HrrA activation seem to have opposing effects on the timing of *hmuO* expression, we asked how these signals are prioritized at the P_*hmuO*_ promoter. To investigate the impact of both regulators on the activation of the heme utilization system, we developed a second mathematical model that focuses on HrrSA and DtxR as main regulators of the P_*hmuO*_ und P_*hrrA*_ activity (Fig. 7A). Like before, in this model the description of the non‐cognate two‐component system (ChrSA) was limited to the cross‐phosphorylation of ChrS on HrrA. In addition, we made the simplifying assumption that the activation of DtxR is proportional to the internal heme levels, based on the fact that the iron availability is proportional to the conversion of the internal heme pool under our experimental (iron‐limiting) conditions. Activated DtxR and phosphorylated HrrA bind to both P_*hmuO*_ and P_*hrrA*_ promoters, where they repress and activate gene expression respectively. Ultimately, increased production of the heme oxygenase HmuO contributes to heme consumption (Frunzke *et al.*, 2011) (see Supplementary Text; Model equations M2 for all details).

Simulations of this computational model revealed a biphasic induction pattern of the P_*hmuO*_ promoter with a quick activation within the first hour after heme addition, followed by a significantly delayed expression boost at approx. 4–5 h (for 4 μM heme) (Fig. 7B), very similar to the experimental dynamics observed before (Fig. 1). The initial activation of P_*hmuO*_ within the model is induced by an instantaneous increase in HrrA~P levels (Fig. 7D) in response to external heme. Since the dynamics of the active form DtxR~Fe^2+^ indirectly depends on the increase in cytoplasmic heme (which is converted to intracellular iron) (Fig. 2C), the DtxR‐mediated repression of P_*hmuO*_, which counteracts the promoter activation by HrrA~P, initiates not before DtxR~Fe^2+^ exceeds a certain threshold – approximately after 1 h – in the model (Fig. 7D). However, low basal levels of endogenously synthesized heme, which are not reflected in the mathematical model, as well as trace amounts of iron (resulting from the pre‐cultivation of the cells, see Experimental Procedures) probably lead to a basal level of activated DtxR in the experimental scenario. These basal levels of DtxR might reduce the initial activation of HrrA~P on P_*hmuO*_ *in vivo*, leading to a less‐pronounced initial increase and a more prominent plateau in the experimental data (Fig. 7C) of the P_*hmuO*_‐*eyfp* reporter output compared to the model prediction (Fig. 7B). Interestingly, when lowering the initial heme concentration, the model predicts an earlier onset of P_*hmuO*_ activation. Strikingly, when experimentally tuning the initial heme levels, we found this exact hierarchy in the activation of the heme utilization module (Fig 7C): A short delay of 3 h at 1 µM heme and a longer delay of 5 h at 4 µM heme.

**Figure 8:**
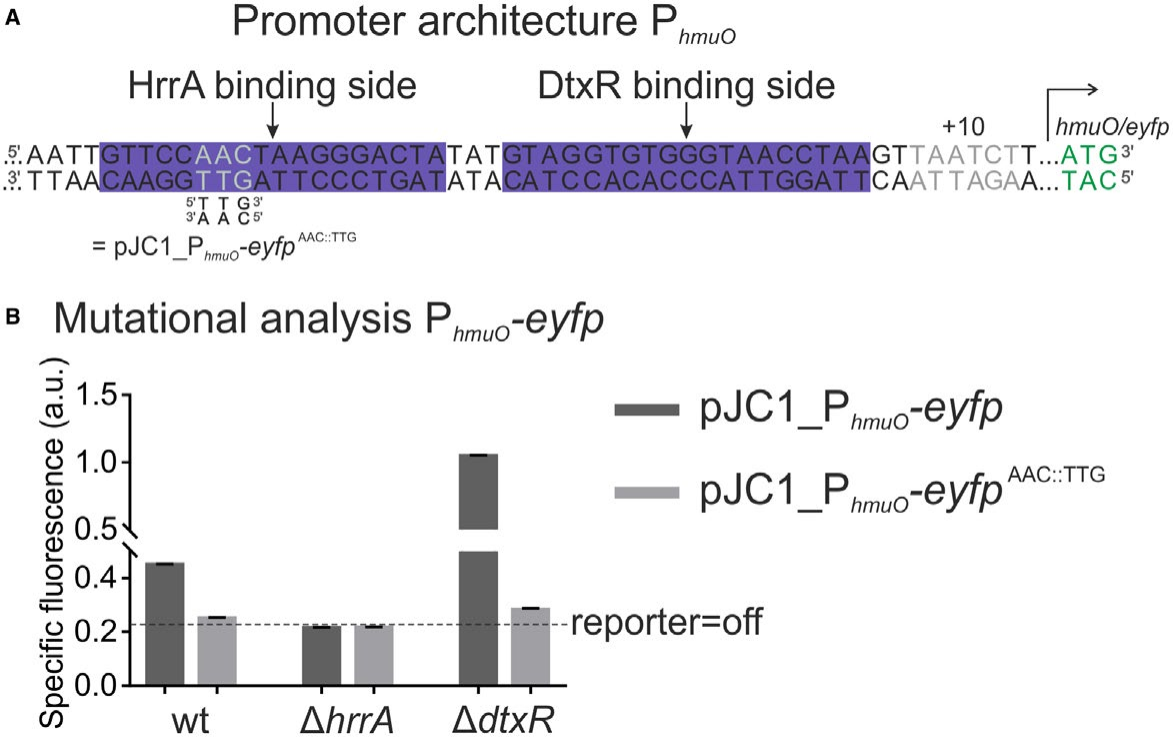
HrrA and DtxR cooperate to control *hmuO* expression in response to iron and heme availability. A. Promoter architecture of *hmuO*. The DtxR binding site was published previously (Wennerhold and Bott, 2006). The AAC::TTG mutation (grey letters) was shown to disrupt HrrA binding to P_*hmuO*_ in vitro (Fig. S3). B. Mutational analysis of P_*hmuO*_. HrrA binding was abolished by introducing the AAC:TTG mutation into the P_*hmuO*_‐*eyfp* reporter. All strains were grown in BHI complex medium supplemented with 4 µM hemin as the Δ*dtxR *strain grows poorly in CGXII medium. After iron starvation overnight, the three strains (wild type, Δ*hrrA* and Δ*dtxR*) were inoculated in BHI and the specific fluorescence (eYFP‐fluorescence/backscatter) was recorded in 15 min intervals. The graph shows the specific fluorescence after 20 h. Full reporter outputs are shown in the supplementary Fig. S4. [Colour figure can be viewed at wileyonlinelibrary.com]

As such, this biphasic induction of *hmuO* strikingly differs from *hrtBA* activation by ChrSA and is governed by the influence of the iron repressor DtxR on *hmuO* expression (Fig. 8). Upon depletion of internal heme (and thus iron‐levels) below a critical threshold, DtxR dissociates from its target promoters and allows their activation (Fig. 7B and D). Accordingly, lower initial heme concentrations within the medium do also shift the time point of heme exhaustion to earlier times, thereby rationalizing the earlier induction of gene expression in the heme utilization module. From a physiological perspective, this again seems very plausible, suggesting that high levels of the heme utilization system are only required under iron limitation when external heme sources are available.

Regarding the regulatory hierarchy of HrrA and DtxR on P_*hmuO*_ activity, one can think of two potential scenarios: either (i) HrrA is a *bona fide* activator of *hmuO* expression or (ii) HrrA‐binding competes with the binding of the repressor DtxR thereby acting as an activator by derepressing *hmuO*, as might be suggested by the close proximity of their binding sites within the P_*hmuO*_ promoter (Fig. 8A). Altogether, our experimental data are clearly in favor for HrrA acting as a *bona fide* activator. While P_*hmuO*_ activity is completely abolished in a *hrrA* mutant (twofold lower reporter output that the WT), it is strongly increased in mutant lacking the *dtxR* gene (Fig. 8B). Furthermore, we tested different triplet mutations in the HrrA binding site in their ability to disrupt HrrA binding. In EMSA‐studies, an AAC::TTG mutation in the middle of the HrrA operator completely abolished *in vitro* binding of HrrA to P_*hmuO*_ (Fig. S3). Subsequently, this mutation was inserted to the pJC1_P_*hmuO*_‐*eyfp* reporter, resulting in the pJC1_P_*hmuO*_‐*eyfp* ^AAC::TTG ^plasmid (Fig. 8A). Irrespective of whether it was introduced to the wild‐type strain or the ∆*dtxR *strain, disruption of HrrA binding decreased the activity of the P_*hmuO*_ reporter to background levels (Figs. 8B and S4). To further validate our findings, we tested the wild type strain carrying the P_*hmuO*_ reporter under different heme and iron conditions (high‐iron/low‐heme; low‐iron/high‐heme, etc). As also revealed by our model (Fig. 7B and C), the results shown in Figure S8 indicate the intimate link between the iron and the heme pool. Due to the basal expression of the heme oxygenase (see Fig. 1 or Fig. 8, WT versus Δ*hrrA*) addition of heme will also impact the bioavailable iron pool, thereby affecting DtxR‐mediated repression of *hmuO*. Thus, according to our current model, HrrA represents an essential activator of *hmuO,* which is required for a basal level of *hmuO* expression (and likely also required for the turnover of the endogenously synthesized heme). DtxR repression places an additional threshold on *hmuO* activation, which is only released when the intracellular iron pool is depleting. Then DtxR dissociates from the *hmuO* promoter allowing full activation by HrrA.

## Discussion

Orchestration of heme homeostasis and detoxification of excess heme appears to be of utmost importance for *Corynebacteriaceae*, as remarkably, several species dedicate two paralogous TCS to this regulatory task (Bott and Brocker, 2012). Here, we provide comprehensive insights into the temporal dynamics of *hmuO* and *hrtBA* expression modulated by HrrSA and ChrSA in *C. glutamicum*. Similar regulatory setups, with two paralogous, exclusively heme‐dedicated systems are, for example, found in *C. diphtheria, Corynebacterium pseudotuberculosis* and *Corynebacterium lipophiloflavum* (Trost *et al.*, 2010; Bott and Brocker, 2012), where the corresponding sequence identities and a conserved genomic synteny suggest a similar role of these systems in the coordinated control of heme detoxification and utilization (Bibb *et al.*, 2007; Bibb and Schmitt, 2010). To our knowledge, these systems represent the first example studied in detail, where two paralogous systems coordinate a complex physiological response by perceiving the same stimulus: heme.

As a general principle, the integrative signaling pathway design appears to be especially beneficial if a multifaceted stimulus requires different regulatory and appropriately timed outputs. The response of *C. glutamicum* to extracellular heme sets an interesting example for this, as the physiological adaption is shaped by a fast‐reacting ChrSA system‐regulating expression of the detoxification module and the HrrSA system contributing to a layered dynamic regulation of heme utilization. In a recent study, we could show that the HKs of both TCS, HrrS and ChrS, slightly differ regarding their responsiveness and their general mode of heme‐protein interaction (Keppel *et al.*, 2018). One obvious difference is, for example, a higher basal activity of the HrrSA system as reflected by the significantly higher output of a *hmuO* reporter compared to *hrtBA* in cells grown on FeSO_4_. This basal activation is likely triggered by the endogenously synthesized heme pool. In contrast to ChrSA, which is required to counteract toxic heme levels, HrrSA has an important role in maintaining heme homeostasis by balancing the synthesis of heme proteins, heme degradation and heme biosynthesis (Frunzke *et al.*, 2011; Hentschel *et al.*, 2014). In this context, it becomes apparent that this system is sensitive toward endogenous heme levels of the cells.

However, kinase activity is not the only factor influencing differential target gene activation. While the P_*chrSA*_ promoter and the divergently located P_*hrtBA*_ promoter are positively autoregulated from one‐centered ChrA binding site (Fig. 3A), *hrrS* and *hrrA* are controlled from two distinct promoters. For *hrrA* as well as *hmuO,* the global iron regulator DtxR adds an additional regulatory layer, thereby integrating information on general iron availability in the cell (Wennerhold and Bott, 2006; Frunzke *et al.*, 2011). Experiments under different iron and heme conditions, furthermore, illustrated the intimate link between these cellular pools (Fig. S8), which is based on the activity of the heme oxygenase – degrading heme to release iron. Here, we could show that HrrA does not simply displace DtxR on the promoter of *hmuO* but is in fact an essential activator, also in the absence of DtxR repression (Fig. 8B). By this means, information on heme (stimulus of HrrS) and Fe^2+^ (co‐repressor of DtxR) is directly integrated at the level of *hmuO* expression and RR (HrrA) synthesis.

In contrast, ChrSA‐mediated activation of *hrtBA* expression is solely influenced by heme availability, as expected for a detoxification system. Here, our data suggested an instantaneous saturation of the ChrS kinase and strong activation of P_*hrtBA*_ in response to exogenous heme – independent of the applied heme concentrations tested in our setup (Fig. 3). Our model and experiments revealed that the overall strength of *hrtBA* expression was determined solely by the duration of the response, as governed by the timescale of heme exhaustion in the medium. Thus, it seems that the comparable levels of P_*hrtBA*_ promoter occupancy independent from stimulus strength ensure an effective detoxification response even for low‐toxic heme concentrations, as claimed for a detoxification module.

Another important factor in the maintenance of intracellular heme homeostasis is the previously reported cross‐phosphorylation between HrrSA and ChrSA (Hentschel *et al.*, 2014). While deletion of *chrS* did not significantly influence the P_*hmuO*_ activation pattern in our reporter studies (Fig. 1B), deletion of *hrrS* resulted in a delayed P_*hrtBA*_ activation in response to heme (Fig. 5). These findings suggest a role of HrrSA as a ‘kick‐start’ system of *chrSA*, thereby giving *C. glutamicum* a competitive edge by shortening the reaction time to mount the detoxification response. Furthermore, the bifunctionality of ChrS ensures efficient proof‐reading of ChrA~P counteracting cross‐phosphorylation by HrrS under non‐inducing conditions where ChrS is dominantly in its phosphatase state.

Physiologically relevant cross phosphorylation between TCSs was, for example, shown in *Bacillus anthracis* (Mike *et al.*, 2014). In this case, the heme responsive HssRS system was shown to cross interact with HitRS, which is activated by cell envelope stress. Cross‐regulation between HssRS and HitRS thereby enables an integrated response of *B. anthracis* to heme and to heme‐induced cell envelope damage (Mike *et al.*, 2014). Interestingly, HrrSA and ChrSA as well as HssRS and HitRS are among the closest‐related TCS in the particular species reflecting that duplication and subsequent specialization represents an evolutionary driver of TCS signaling toward the integration of multiple signals and the creation of a multifaceted response to complex stimuli. A similar scenario is found with the NarPQ and NarXL systems regulating the response to nitrate and nitrite in *Escherichia coli* (Rabin and Stewart, 1993). For these closely related systems, significant cross phosphorylation appears to play a role in the modulation of target gene activation and maintenance of nitrogen homeostasis (Noriega *et al.*, 2010).

The regulatory setup shaping the response to heme may also have considerable impact on heme tolerance of the particular species. Already decades ago, van Heyningen reported on the differential sensitivity of *Bacillus* species to heme (Van Heyningen, 1948). Mike and co‐workers correlated an increased tolerance, as observed for *B. anthracis*, with the employment of two cross‐regulating TCS coordinating heme export (HssRS) and cell envelope stress (HitRS) (Mike *et al.*, 2014). Therefore, it might be conceivable that the employment of two heme‐responsive TCSs by some corynebacterial species enables a more robust control of heme homeostasis compared to the regulation by a single system. Remarkably, while the HrtBA exporter is conserved among many Gram‐positive species, the TCS systems ‘in charge’ do not share significant sequence similarity – especially not in their membrane‐embedded sensor domains (Stauff and Skaar, 2009b; Keppel *et al.*, 2018). This overall scenario of two cross‐regulating TCSs modulating heme homeostasis and detoxification underlines a favorable concept nature employs to respond to the multifaceted stimulus heme.

In summary, we have approached the regulatory interplay between the heme‐responsive HrrSA and ChrSA TCSs of *C. glutamicum* by a comprehensive screening of various mutant strains carrying different target promoters. Generation of a mathematical model based on this data set revealed the underlying mechanisms triggering the antagonistic temporal dynamics in TCS signaling, which shape the cellular response toward the ‘toxic, but tasty’ heme molecule. We believe that the approach of combining time‐resolved monitoring of gene expression and systems‐level modeling of the underlying regulatory networks is key to understanding the logic behind complex homeostatic responses in bacteria.

## Experimental procedures

### Bacterial strains and growth conditions

Bacterial strains used in this study are listed in Table S1. *C. glutamicum* strain ATCC 13032 was used as wild type (Kalinowski *et al.*, 2003) and either cultivated in BHI (brain heart infusion, Difco BHI, BD, Heidelberg, Germany) as complex medium or CGXII (Keilhauer *et al.*, 1993) containing 2% (w/v) glucose as minimal medium. All cultivations were performed at 30°C and, if necessary, 25 μg/ml kanamycin was added to the medium for selection. For standard cloning applications, *E. coli* DH5α was cultivated in Lysogeny Broth (Difco LB, BD, Heidelberg, Germany) medium at 37°C in a rotary shaker and for selection, 50 μg/ml kanamycin was added to the medium.

### Recombinant DNA work and cloning techniques

Standard cloning and other molecular methods were performed according to the standard protocols (Sambrook and Russell, 2001). For most applications, chromosomal DNA of *C. glutamicum* ATCC 13032 was used as a template for PCR amplification of DNA fragments and was prepared as described previously (Eikmanns *et al.*, 1994). All sequencing and synthesis of oligonucleotides was performed by Eurofins Genomics (Ebersberg, Germany). For the construction of plasmids, the DNA regions of interest were amplified from chromosomal *C. glutamicum* DNA with oligonucleotides listed in Table S2 and ligated into the plasmid backbone (see Table S3) via restriction sites indicated in the same table. Genomic integrations and/or deletions were performed using the pK19*mobsacB* plasmid and the two‐step homologues recombination method described earlier (Niebisch and Bott, 2001). Point mutations for the integration of kinase mutants or construction of a mutated reporter plasmid were introduced via ‘QuickChange Lightning’ site‐directed mutagenesis according to the supplier’s manual (Agilent Technologies, Inc., Santa Clara, USA).

### Reporter assays

For reporter studies, *C. glutamicum* wild type or one of the mutant strains were transformed with a reporter plasmid (Table S3). A preculture in BHI medium (25 µg/ml kanamycin) was inoculated from a fresh BHI agar plate and incubated for 8–10 h at 30°C in a rotary shaker. After that, cells were transferred into a second preculture in CGXII medium (Keilhauer *et al.*, 1993) containing 2% (w/v) glucose and 0 µM FeSO_4_ to starve the cells from iron. However, protocatechuic acid (PCA) was present in the preculture, allowing the uptake of trace amounts of iron. After growth overnight, the main culture was inoculated to an OD_600_ of 1 in CGXII medium and cultivated in 48‐well Flowerplates (m2p‐labs GmbH, Aachen, Germany) at 30°C, 95% humidity, 1200 rpm. For the hemin stock solution, hemin (Sigma Aldrich, Munich, Germany) was dissolved in 20 mM NaOH to a concentration of 2.5 mM and, as an iron source, added to the medium in the desired concentrations. Growth of the cells (biomass production) was recorded as the backscattered light intensity of sent light with a wavelength of 620 nm (signal gain factor of 12). For the measurement of eYFP fluorescence, the culture was excited at 510 nm and emission was measured at 532 nm (signal gain factor of 50). Measurements were performed in 15 min intervals.

### Electrophoretic mobility shift assays

To characterize the operator sequence of HrrA, the protein was produced in *E. coli* BL21 and purified as His_6_‐tagged variant from cells as described previously (Frunzke *et al.*, 2011). As ligand, 30 bp DNA fragments with triplet mutations were amplified and subsequently, 100 ng of the fragments were incubated with 0×, 10× and 30× excess of HrrA in in EMSA buffer (250 mM Tris–HCl pH 7.5, 25 mM MgCl_2_, 200 mM KCl, 25% (v/v) glycerol). After 30 min at room temperature, the samples were loaded to a native 12% polyacrylamide gel (TBE‐based, TBE (89 mM Tris base, 89 mM boric acid, 2 mM Na_2_EDTA, loading dye: 0.01% (w/v) xylene cyanol dye, 0.01% (w/v) bromophenol blue dye, 20% (v/v) glycerol, 1 × TBE). Electrophoresis was carried out for 60 min at 160 V. DNA was subsequently stained with SYBR Green I (Sigma Aldrich, Munich, Germany).

### Measurement of cell‐associated heme levels

The protocol for the fluorescent measurement of (total) cell‐associated heme was derived from an assay described by Sassa (1976). A preculture containing *C. glutamicum* wild‐type cells was inoculated in BHI medium from a fresh BHI agar plate and incubated for 8–10 h at 30°C in a rotary shaker. After that, cells were transferred into a second preculture in CGXII medium containing 2% (w/v) glucose and 0 µM FeSO_4_ to starve the cells from iron. After growth for 12–16 h, the main culture was inoculated to an optical density (OD_600_) of 1 in CGXII medium. Before addition of 4 µM hemin, as well as 5 min, 30 min, 1, 3, 6, 7 and 23 h after hemin addition, samples were taken for the determination of cell‐associated heme. For that, cells corresponding to an OD_600 _of 2 in 250 µl were harvested and washed once in 250 µl PBS. Subsequently, the pellet was resuspended in 250 µl 20 mM oxalic acid and stored at 4°C for max 5–6 h. Then, 250 µl of 2 M oxalic acid were added and each sample was heated to 98°C for 30 min. For the measurement, 200 μL of each sample were transferred to a 96‐well microtiter plate and the fluorescence was recorded on a Tecan (Tecan Trading AG, Switzerland) Microplate Reader (excitation at 400 nm and emission at 608 nm). The values for each sample were normalized to the emission of an unheated control sample (cells before hemin addition in 1 M oxalic acid).

### Mathematical models and mutant simulation

Two mathematical models were developed to assess the determining factors of the dynamics of the heme detoxification and utilization modules (see Supplementary Text; Model equations M1 and M2). A set of ordinary differential equations (ODEs) describes the time‐dependent changes in the different components of the two networks under varying heme concentrations as stimulus of both systems. The interactions between the kinases and the response regulators of the two TCSs were described based on the modeling approach by Groban and co‐workers (2009), while thermodynamic modeling (Bintu *et al.*, 2005) was used to describe the target gene regulation in both systems. The dynamics of the individual components were simulated for wild‐type conditions as well as different mutant strains. The numerical solution of the ODEs as well as the individual simulations were performed with MATLAB^TM^ software (The MathWorks, Inc.).

## Conflict of interest

The authors declare that the research was conducted in the absence of any commercial or financial relationships that could be construed as a potential conflict of interest.

## Author contributions

MK, HP, GF and JF conceived the study; MK and CG performed the experiments; MK, HP, GF and JF analyzed the data; HP and GF generated the models;MK, HP, GF and JF wrote the manuscript.
