# Supplementary material for: Toxic but tasty – temporal dynamics and network architecture of heme‐responsive two‐component signaling in Corynebacterium glutamicum
Source: Mol Microbiol. 2019 Mar 22;111(5):1367–81. doi: 10.1111/mmi.14226 (PMC6850329; doi:10.1111/mmi.14226)
Supplement: Supplementary file 1 [file MMI-111-1367-s001.docx]

Supplementary Information

**Toxic but tasty - Temporal dynamics and network architecture of heme-responsive two-component signalling in *Corynebacterium glutamicum***

Marc Keppel^1#^, Hannah Piepenbreier^2#^, Cornelia Gätgens^1^, Georg Fritz^2^* and Julia Frunzke^1^*

^1^Institute of Bio- und Geosciences, IBG-1: Biotechnology, Forschungszentrum Jülich, 52425 Jülich, Germany

^2^LOEWE-Zentrum für Synthetische Mikrobiologie, Philipps-Universität Marburg, 35032 Marburg, Germany

*Corresponding authors:

Georg Fritz; Email: [georg.fritz@synmikro.uni-marburg.de](mailto:georg.fritz@synmikro.uni-marburg.de); Phone: +49 6421 28 22582

Julia Frunzke; Email: [j.frunzke@fz-juelich.de](mailto:j.frunzke@fz-juelich.de); Phone: +49 2461 615430

^#^These authors contributed equally to this work.

Content

**Figure S1:** Growth curves after application of additional heme pulses.

**Figure S2:** Additional heme pulses do not prime P*_hrtBA_.*

**Figure S3:** Electrophoretic Mobility Shift Assays (EMSAs) reveal crucial nucleotides for HrrA binding to the operator.

**Figure S4:** Control of P*_hmuO_* by HrrA and DtxR.

**Figure S5**: Dephosphorylation of the response regulator ChrA determines the dynamics of the target gene activation in the wildtype and a *chrSQ191A* phosphatase mutant.

**Figure S6:** The *in vitro* data suggest a cross-phosphatase activity of HrrS which did not result in a model that quantitatively fits to the behavior of the Δ*chrS* mutant *in vivo* data

**Figure S7:** An increased phosphatase activity of HrrS prevents a delayed P*_hmuO_* activation by HrrA~P.

**Figure S8:** P*_hmuO_*-*eyfp* screening under different environmental conditions.

**General supplement file S1:** In depth description of the mathematical models and equations

**Table S1:** Bacterial strains used in this study.

**Table S2:** Oligonucleotides used in this study.

**Table S3:** Plasmids used in this study.

**Table S4:** Parameters used in the mathematical model of the *C. glutamicum* heme detoxification module.

**Table S5:** Additional parameters used in the mathematical model of the *C. glutamicum* heme utilization module.

**Table S6**: Transformation of the units within the mathematical models.

| **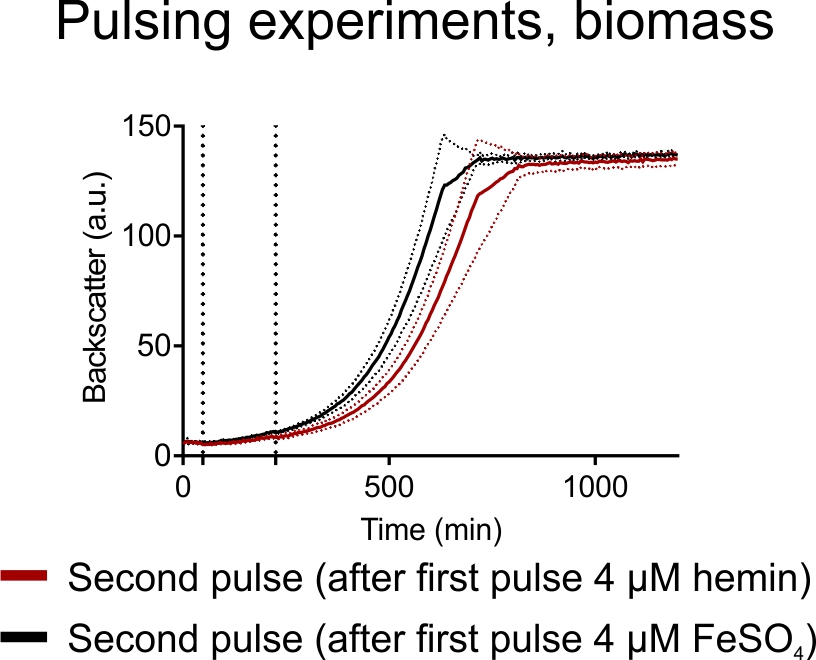** |
| --- |
| **Figure S1: Growth curves after application of additional heme pulses**  *C. glutamicum* cells were transformed with the target gene reporter pJC1_P*_hrtBA_*-*eyfp* and starved from iron overnight as described in material and methods. Subsequently, the cells were inoculated in CGXII minimal medium with 2% (w/v) glucose containing no iron source and transferred to the microbioreactor system (Biolector) where eYfp fluorescence (=reporter output) and backscatter (biomass) was measured in 5 minutes intervals. After 45 min, hemin (red line) or FeSO_4_ (black line) was added to a final concentration of 4 µM. A second pulse of 4 µM hemin was applied to both cultures after 225 min. |

| **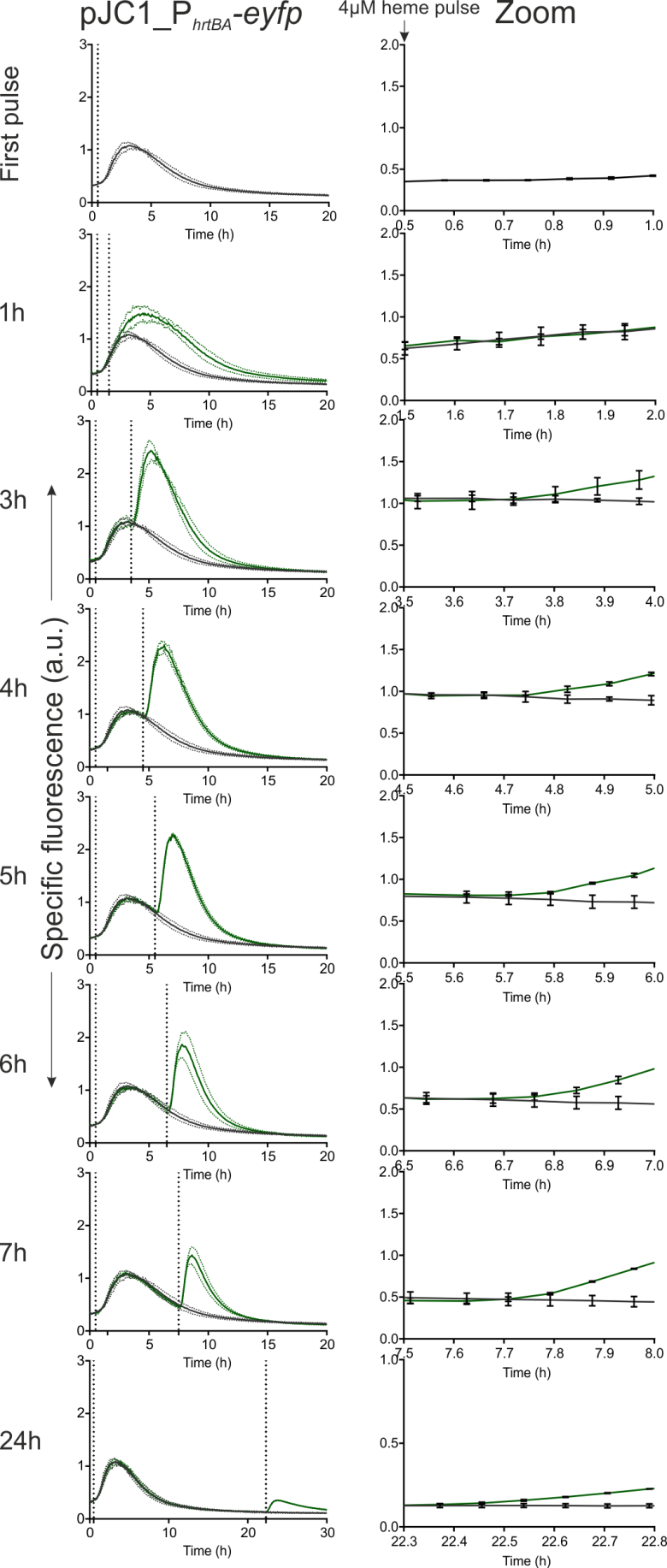** |
| --- |
| **Figure S2: Additional heme pulses do not prime P*_hrtBA_.***  *C. glutamicum* cells were transformed with the target gene reporter pJC1_P*_hrtBA_*-*eyfp* and starved from iron overnight as described in material and methods. Subsequently, the cells were inoculated in CGXII minimal medium with 2% (w/v) glucose containing no iron source and transferred to the microbioreactor system (Biolector) where eYfp fluorescence (=reporter output) and backscatter (biomass) was measured in 5 minutes intervals. After 30 min, hemin was added to a final concentration of 4 µM. A second pulse of 4 µM hemin (resulting in a final hemin concentration of 8 µM) was applied after 1h, 3h, 4h 5h, 6h, 7h or 24h, respectively. |

| *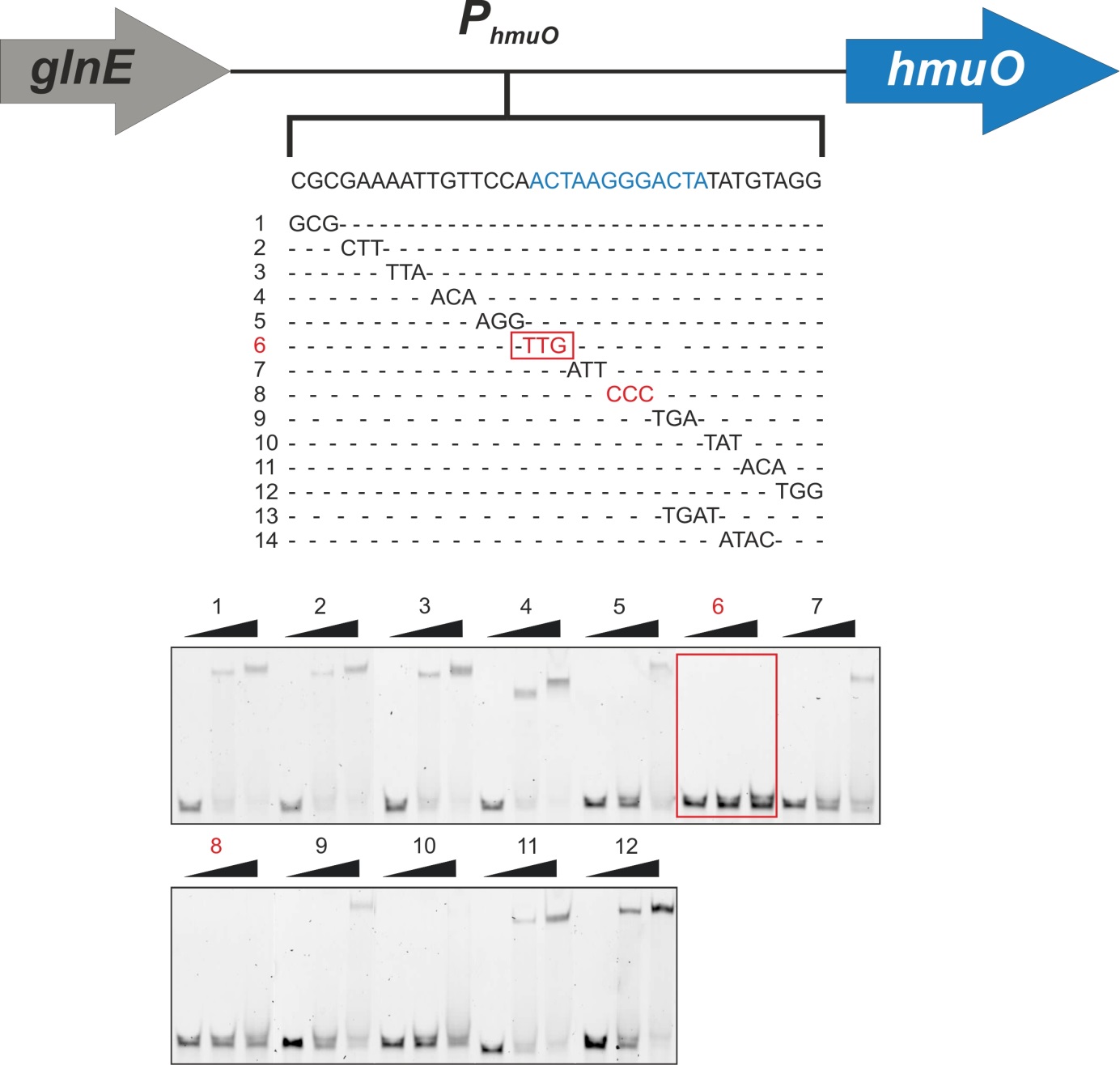* |
| --- |
| **Figure S3: Electrophoretic Mobility Shift Assays (EMSAs) reveal crucial nucleotides for HrrA binding to the operator.**  For all EMSAs, 36 Bp of mutated DNA fragments (100 ng) were used and purified HrrA-His protein was applied in 0, 10 and 30-fold molecular excess for each sample. Mutation 6 (ACT::TTG) and mutation 8 (GGG::CCC) led to strongly reduced binding of HrrA to P*_hmuO_* and similar P*_hmuO_-eyfp* output like observed in a ∆*hrrA* deletion mutant (Fig. S5B). |

| **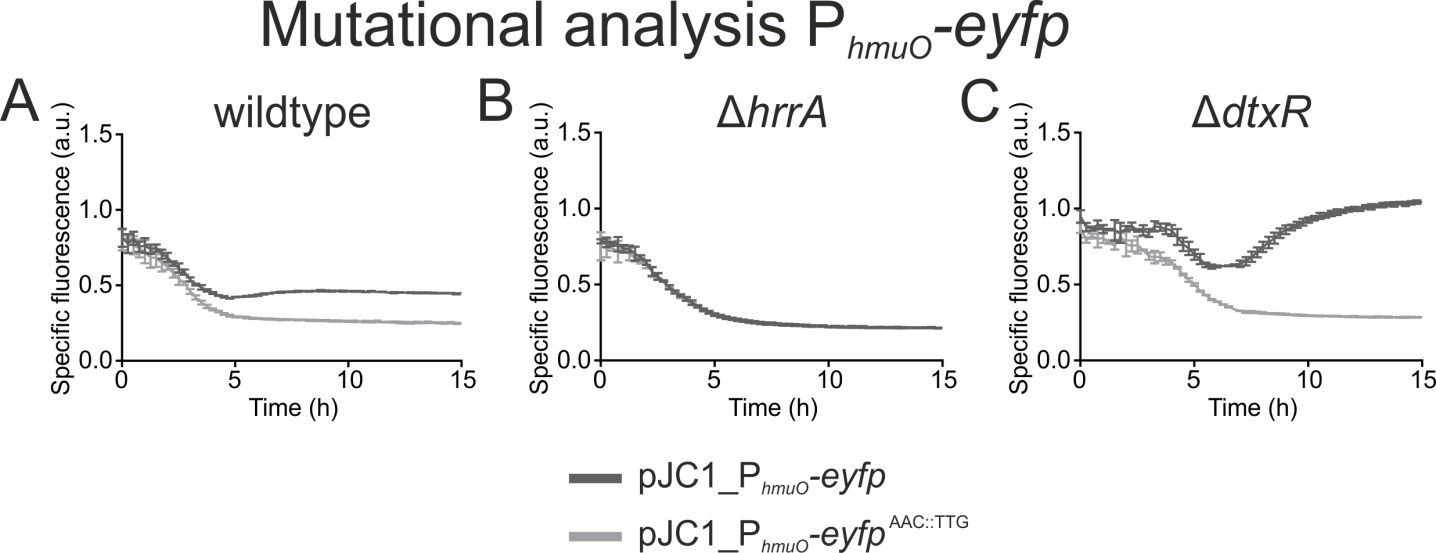** |
| --- |
| **Figure S4: Control of P*_hmuO_* by HrrA and DtxR.**  HrrA binding was abolished by introducing the AAC::TTG mutation into the P*_hmuO_-eyfp* reporter. After iron starvation overnight, the three strains (A: wildtype, B: Δ*hrrA* and C: Δ*dtxR*) were inoculated in BHI complex medium supplemented with 4 µM hemin and the specific fluorescence (eYFP-fluorescence/backscatter) was recorded in 15 minutes intervals. The strains were grown in BHI complex medium as the Δ*dtxR* strain grows poorly in CGXII medium.  **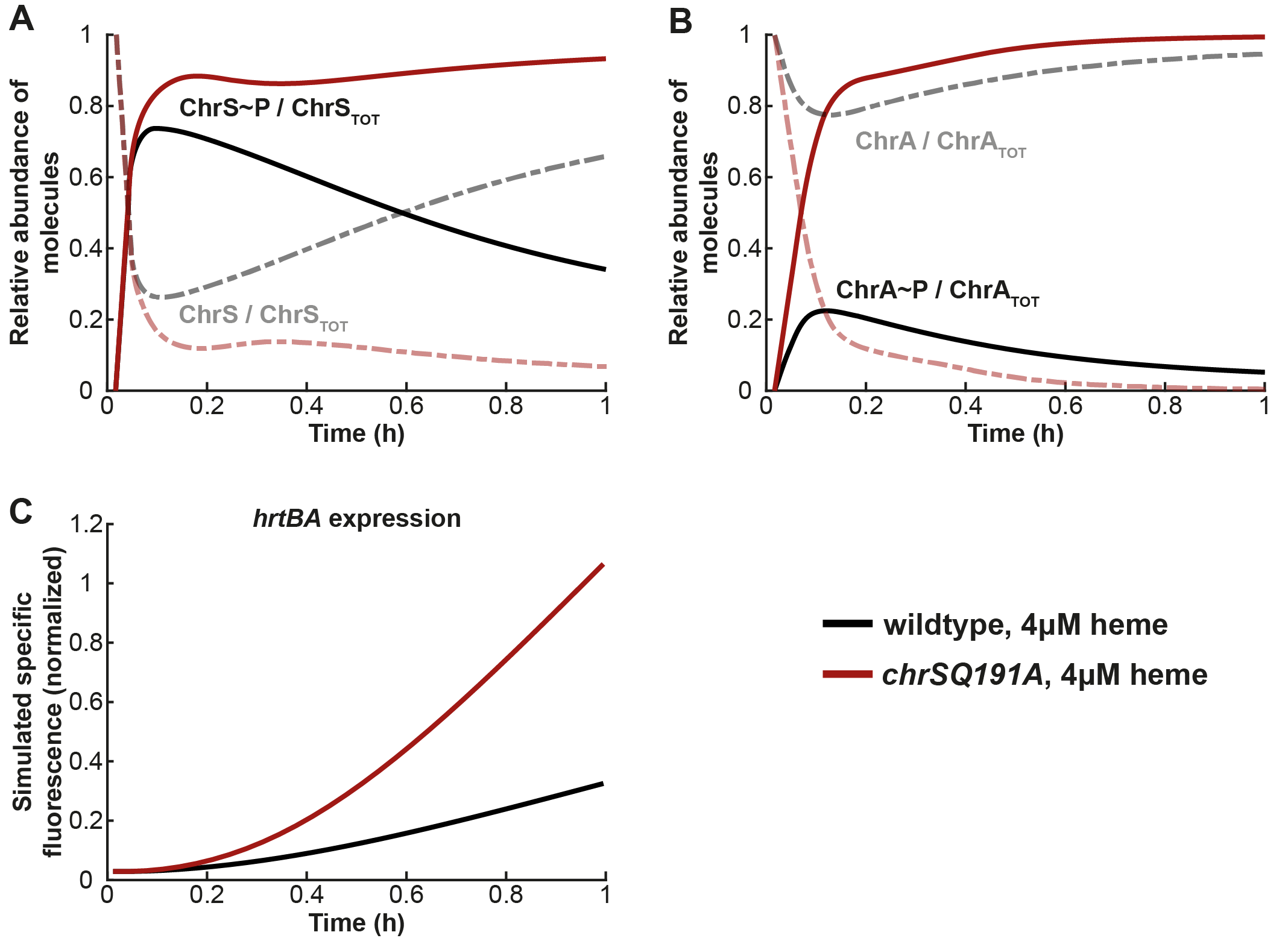**  **Figure S5: Dephosphorylation of the response regulator ChrA determines the dynamics of the target gene activation in the wildtype and a *chrSQ191A* phosphatase mutant.**  The activation of the P*_hrtBA_-eyfp* reporter critically hinges on the levels of phosphorylated response regulator ChrA~P in the wild type (black line) and a *chrSQ191A* phosphatase mutant (dark red line). (A) Initial high levels of external heme autophosphorylate and thereby activate the histidine kinase ChrS strongly in the wild type as well as the *chrSQ191A* phosphatase mutant, leading to a significant portion of the phosphorylated form of ChrS (ChrS~P/ChrS_TOT_$\approx$ 0.8, solid line). But immediately after incipient stimulus decline, the balance between ChrS~P and ChrS shifts towards the non-phosphorylated form ChrS (ChrS/ChrS_TOT_, dashed line) in the wild type. However, the levels of ChrS~P stay high in the *chrSQ191A* phosphatase mutant. (B) The activation of the response regulator ChrA depends on the state of the histidine kinase ChrS. Phosphorylated and thereby activated ChrS (ChrS~P) is able to phosphorylate and activate ChrA (ChrA~P), while the non-phosphorylated form of ChrS (ChrS) naturally functions as a phosphatase for ChrA. Since the levels of non-phosphorylated ChrS are very low at the very beginning in both strains, phosphatase activity of ChrS has insignificant impact and the levels of ChrA~P are similar (ChrA~P /ChrA_TOT_, solid line). However, the increasing levels of ChrS after heme depletion lead to an immediate dephosphorylation of ChrA~P and in the wild type, while the ChrA~P levels stay high in the *chrSQ191A* phosphatase mutant, leading to a maximal phosphorylation level of ChrA of 25% in the wild type, compared to 100% in the phosphatase mutant. (C) As ChrA~P activates the target promoter P*_hrtBA_*, the significant differences between the ChrA~P levels in the wild type and the *chrSQ191A* phosphatase mutant lead to varying reporter outputs of P*_hrtBA_-eyfp*. While both strains show similar reporter outputs within the first minutes where ChrS~P and thereby ChrA~P levels are identical and phosphatase activity of ChrS plays no significant role, they diverge from the time point of differentiation in ChrA~P levels onwards. |

| 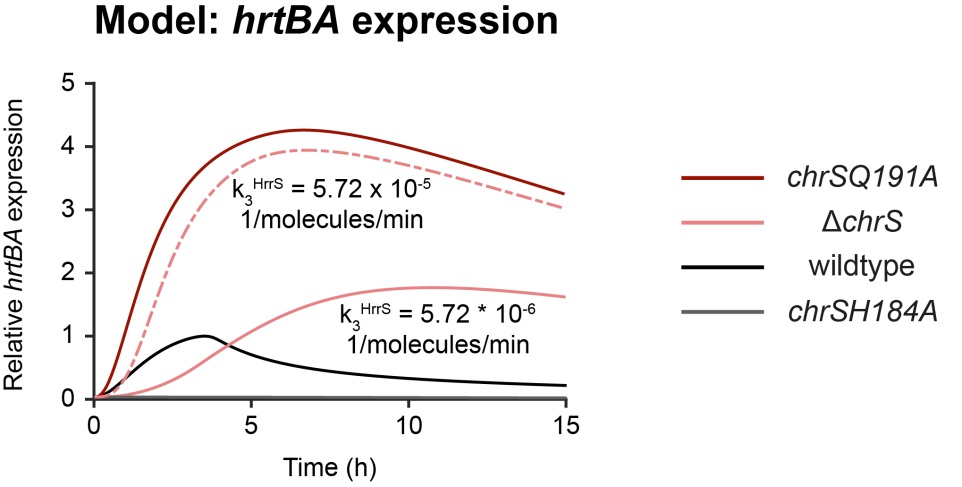 |
| --- |
| **Figure S6: The *in vitro* data suggest a cross-phosphatase activity of HrrS which did not result in a model that quantitatively fits to the behavior of the Δ*chrS* mutant *in vivo* data.**  Setting $k_{3}^{HrrS}$ to 5.7 x 10^-5^ *1/molecules/min* as expected from the *in vitro* data in ([Hentschel et al., 2014](#_ENREF_7)) led to an excessive response of P*_hrtBA_* in the Δ*chrS* mutant (dashed red line)*.* A reduced cross-phosphatase avtivity of HrrS by a factor of 10 reproduces the mutant behaviour quantitatively (solid red line). |

| 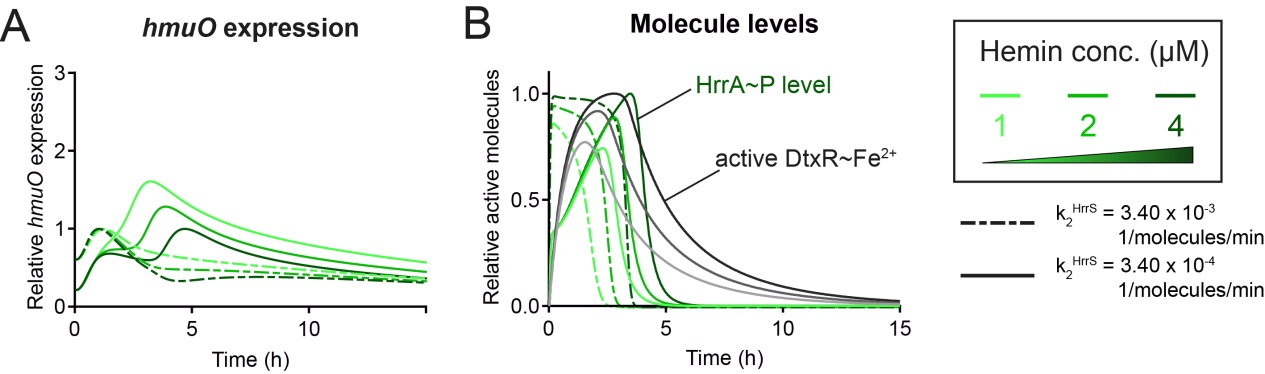 |
| --- |
| **Figure S7: An increased phosphatase activity of HrrS prevents a delayed P*_hmuO_* activation by HrrA~P.**  Setting $k_{2}^{HrrS}$ to 3.40 x 10^-3^ *1/molecules/min* as expected from the *in vitro* data in ([Hentschel et al., 2014](#_ENREF_7)) did not result in a model that quantitatively fits to the behaviour of the *in vivo* data in wild-type. The strong phosphatase activity decreases HrrA~P levels immediately after stimulus reduction and no delayed P*_hmuO_* activation is possible. Decreasing $k_{2}^{HrrS}$ about a factor of 10 improved the ability to reproduce the *in vivo* behaviour. |


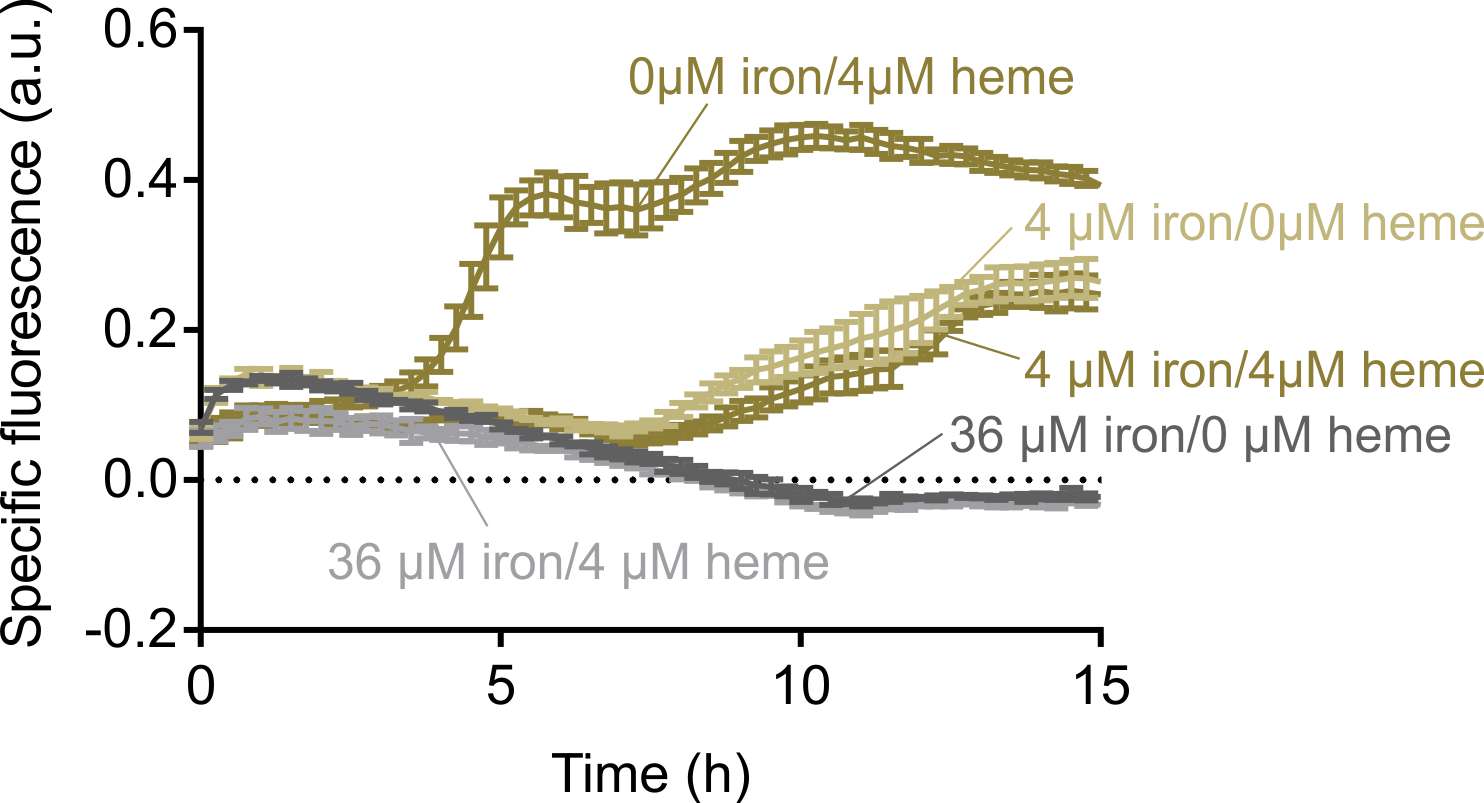


**Figure S8: P*_hmuO_*-*eyfp* screening under different environmental conditions.**

*C. glutamicum* cells carrying the pJC1_P*_hmuO_*-*eyfp* reporter plasmid were iron starved overnight and inoculated in CGXII medium containing FeSO4, hemin or both as iron source in the indicated concentrations. Biomass (backscatter) and fluorescence were measured every 15 minutes and the specific fluorescence was calculated as (eYFP-fluorescence/backscatter) and normalized to an empty well.

These data demonstrate the link between the heme and iron pools in the cells. Due to the basal expression of the heme oxygenase (see Figure 1 or Figure 8, WT versus Δ*hrrA*) addition of heme will also impact the bioavailable iron pool, thereby affecting DtxR activity. According to our current model HrrA represents an essential activator of *hmuO,* which is required for a basal level of *hmuO* expression (and likely also required for the turnover of the endogenously synthesized heme). DtxR repression places an additional treshhold on *hmuO* activation, which is only released when the intracellular iron pool is depleting. Then DtxR dissociates from the *hmuO* promoter allowing full activation by HrrA.

**Description of mathematical models**

The heme detoxification and utilization network contains different layers of regulation that have to be considered within the mathematical model.

**Bacterial growth kinetics**

Heme is used as alternative iron source for growth under iron-starvation conditions. In order to study heme depletion within the growth medium in our experiments, we described the uptake of external heme via the heme importer HmuTUV and the subsequent incorporation of the cytoplasmic heme into biomass via utilization enzymes based on Michaelis-Menten expressions. As a simplifying assumption, we expected a constant maximal velocity for the import (*v_max_^IMP^*) as well as the heme consumption (*v_max_^CON^*) and described the concentrations of the two extra- and intracellular heme pools (*[H_EX_]* and *[H_IN_]*, respectively) as follows:

$$\frac{d\left[ H_{EX} \right]}{dt}= -v_{max}^{IMP}\frac{\left[ H_{EX} \right]}{K_{M}^{IMP}+\left[ H_{EX} \right]}\left[ cells \right]$$

$$\frac{d\left[ H_{IN} \right]}{dt}= v_{max}^{IMP}\frac{\left[ H_{EX} \right]}{K_{M}^{IMP}+\left[ H_{EX} \right]}-v_{max}^{CON}\frac{\left[ H_{IN} \right]}{K_{M}^{CON}+\left[ H_{IN} \right]}-\gamma\left[ H_{IN} \right],$$

while *K_M_^IMP^* and *K_M_^CON^* represent the Michaelis constants. Thus, the bacterial growth was formulated in terms of

$$\frac{d\left[ cells \right]}{dt}=\beta*\frac{\left[ H_{IN} \right]}{K_{M}^{CON}+\left[ H_{IN} \right]}*[cells]$$

with the maximal growth rate *β* that serves as an effective description of heme consumption via diverse enzymatic reactions.

Given the fact that the total content of iron in *C. glutamicum* cells is in a range of 0.3-0.5 mg_FE_/g_CDW_ and 1 g_CDW_ corresponds to approx. $6.5 x {10}^{11}$ cells (Unthan et al., 2014), we calculate an iron demand of a single cell of $\sim5 x {10}^{6}$ Fe^2+^ molecules per cell ($1g_{FE}\sim{10}^{22}$ molecules). Considering a generation time of $\sim$120 minutes in our experiments (see Fig. 2A in the main text), $\sim{4 x 10}^{4}$ molecules of internal heme – as alternative iron source - have to be incorporated into biomass per minute. Taking this estimation into account, our mathematical description of the bacterial growth kinetics predicted a depletion of external heme between 2.5 and 3.5 hours, dependent on the initial concentrations. The cytoplasmic pool of heme is depleted with a time delay of $\sim$2 hours, which then leads to cessation in cell growth (see Fig. 2B,C in the main text). Finally, the total heme pool per cell ([*H^cell^_TOT_*], Fig. 2E), was defined as the sum of cytoplasmic heme per cell and the portion of external heme per cell

$${[H}_{TOT}^{cell}]={[H}_{IN}]+\frac{{[H}_{EX}]}{\left[ cells \right]}.$$

**Stimulus perception and signalling in the two-component systems**

The described overall flux of the stimulus heme through the network as the base for cell growth represents the first layer that was quantified within the model. In addition, the dynamics within the TCSs that sense heme as their stimulus represent a second layer of regulation within the system. According to the reporter assays, we assumed a constitutive expression and thereby production of HrrS, while the phosphorylated response regulator HrrA activates the production of HrrA and HmuO. Besides, ChrS, ChrA and HrtBA production is dependent from ChrA. We expected a production of the non-phosphorylated form exclusively, both in case of the histidine kinases as well as the response regulators. Given that no information is available about the copy number of the heme importer HmuTUV, we assumed a constant number of transporter molecules independent from time and hemin levels. Both kinases, HrrS and ChrS, phosphorylate their cognate and non-cognate response regulator ([Hentschel et al., 2014](#_ENREF_7)) in response to external heme as their stimulus ([Keppel, Davoudi, Gätgens, & Frunzke, 2018](#_ENREF_9)). In the following, we will give the quantification of different reactions for ChrSA as an example but the description is identical for HrrSA. Following this approach of Groban and co-workers ([Groban, Clarke, Salis, Miller, & Voigt, 2009](#_ENREF_6)), we described the transition from the non-phosphorylated form into the phosphorylated one of the histidine kinases and the response regulators but did not quantify the phosphotransfer in detail. Thus, we expected the autophosphorylation of the kinases (ChrS) in response to stimulus perception

| $ChrS\underset{\to}{H_{EX}}ChrS\sim P$ |  |
| --- | --- |

and described the reaction by

| $\frac{d\left[ ChrS\sim P \right]}{dt}=I_{ChrS}\left( H_{EX} \right) \left[ ChrS \right],$ |  |
| --- | --- |

while $I_{ChrS}\left( H_{EX} \right)=k_{+}^{ChrS}*\frac{\left[ H_{EX} \right]}{K_{H_{EX}}+\left[ H_{EX} \right]}$describes the autophosphorylation rate, dependent on the external heme concentration. The autophosphorylation threshold is given by *K_HEX_*, while *k_+_^ChrS^* determine the speed of the autophosphorylation reaction. *[ChrS]* and *[ChrS~P]* describe the concentration of the phosphorylated or non-phosphorylated form of the kinase.

A phosphorylated kinase (ChrS~P) can donate its phosphate to the response regulator

| $ChrS\sim P+ChrA \to ChrS+ChrA\sim P.$ |  |
| --- | --- |

During the reverse process of dephosphorylation of the response regulator, the phosphate is not passed back to the kinase

| $ChrS+ChrA\sim P \to ChrS+ChrA.$ |  |
| --- | --- |

The phosphorylation and dephosphorylation of ChrA was expected to follow second order kinetics, which were quantified by the rate constants *k_i_*:

| $\frac{d\left[ ChrA\sim P \right]}{dt}=k_{1}^{ChrS}*\left[ ChrS\sim P \right]*\left[ ChrA \right]-k_{2}^{ChrS}*\left[ ChrS \right]*[ChrA\sim P]$ |  |
| --- | --- |

The phosphotransfer from *ChrS~P* to *ChrA* is determined by the rate constant *k_1_^ChrS^*, while the reverse reaction (*ChrS* dephosphorylates *ChrA~P*) occurs with a rate dependent on the rate constant *k_2_^ChrS^*. Cross-phosphorylation of both kinases to their non-cognate response regulators was expected, cross-dephosphorylation could not be observed previously and was therefore not included into the model.

**Regulatory dynamics of target genes**

The target gene activation of the phosphorylated response regulators represents the third layer of regulation and a mathematical description based on thermodynamic modelling quantified the observed transcriptional regulation. Following the approach of Bintu and co-workers ([Bintu et al., 2005](#_ENREF_1)), the activation of gene expression from the P*_hrtBA_* and the P*_chrSA_* promoter by phosphorylated ChrA could then be formulated in terms of *ChrA~P* concentrations, such that the dynamic equation for the HrtBA protein levels, *[HrtBA]*, read:

| $\frac{d\left[ HrtBA \right]}{dt}=\alpha* \left( \frac{1+\omega\left( \frac{\left[ ChrA\sim P \right]}{\kappa} \right)^{n}}{1+\left( \frac{\left[ ChrA\sim P \right]}{\kappa} \right)^{n}} \right)-\gamma*[HrtBA]$ |  |
| --- | --- |

We assumed a basal protein production with an effective rate *α* and dilution with a rate proportional to *γ* for all components within the systems according to growth. The basal production of the proteins is a combined representation of the processes of transcription and translation, justified by the fact that e.g. mRNA maturation and degradation proceed on much faster time-scales than the signalling and target gene regulation within the system and were therefore not relevant for the investigated dynamics. The ratio of maximal to basal promoter activity is defined as the fold-change *ω* ([Bintu et al., 2005](#_ENREF_1)). *Κ* in turn represents a measurement of the concentration of phosphorylated response regulator ChrA at which P*_hrtBA_* is activated and the hill coefficient *n* reflects all forms of cooperativity in ChrA binding to the promoter. According to the fact that we based our mathematic model on the experimental data of the performed reporter assays, we discriminated within our model between the proteins of the systems itself and the reporter output that reflects the production of detectable fluorescence proteins based on the original promoter activity. To this end, we formulated one equation for the regulated protein production and one equation for the corresponding YFP production each and integrated an effective parameter for YFP bleaching and degradation processes in the latter equation. The dynamic equations for all components can be found in M1 and M2.

**Model parameters**

In order to calibrate the model, various parameters concerning the cell growth as well as the dynamics within the TCSs could be fixed to their physiological values based on experimental data. The remaining ones were adjusted within physiological intervals to reproduce the experimental data of promoter activity within the mathematical model (for further descriptions see Tables S4 and S5 and Fig. S5/S6).

**Mutant simulations**

For the purpose of predicting the behaviour of several mutants within the model of the heme detoxification module, we adapted the individual parameters of the mathematic equations to the experimentally given scenarios. For the wild type, we simulated the time-dependent dynamics based on the complete set of parameters we fixed within our model. To knock out a protein in the model, we set the participating rate constants of the protein-based reactions as well as the initial concentrations to zero. In case of the Δ*chrS* mutant, we set the basal concentration *ChrS_INI_* as well as the rate constants for phosphorylation (*k_1_^ChrS^*) and dephosphorylation (*k_2_^ChrS^*) to zero. Within the phosphatase mutant *chrSQ191A,* the dephosphorylation step of ChrA is not possible. Thus, we exclusively chose *k_2_^ChrS^* to zero. In contrast, inhibition of ChrS kinase activity (*chrSH186A*) leads to a lack in the ability of autophosphorylation of ChrS and thereby the option of phosphotransfer to the response regulator ChrA. Disrupting the autophosphorylation of ChrS could be simulated by setting *k_+_^ChrS^* to zero. In addition, the lack of phosphotransfer could be quantified by *k_1_^ChrS^* = 0.

**Model equations M1:** ODEs of the mathematical model of the *C. glutamicum* heme detoxification module

Variables within the model:

| **Name** | **Description** |
| --- | --- |
| *H_EX_* | External heme |
| *H_IN_* | Internal heme |
| *cells* | Cells of *C. glutamicum* |
| *ChrS* | Unphosphorylated histidine kinase ChrS |
| *ChrS~P* | Autophosphorylated histidine kinase ChrS |
| *HrrS* | Unphosphorylated histidine kinase HrrS |
| *HrrS~P* | Autophosphorylated histidine kinase HrrS |
| *ChrA* | Unphosphorylated response regulator ChrA |
| *ChrA~P* | Phosphorylated response regulator ChrA |
| *HrtBA* | Heme exporter HrtBA |
| *ChrS_TOT_-YFP* | YFP proteins corresponding to the total amount of the kinase ChrS (The production is under the control of P*_chrSA_* promoter) |
| *ChrA_TOT_-YFP* | YFP proteins corresponding to the total amount of the response regulator ChrA (The production is under the control of P*_chrSA_* promoter) |
| *HrtBA-YFP* | YFP proteins corresponding to the total amount of the heme exporter HrtBA (The production is under the control of P*_hrtBA_* promoter) |

ODEs:

| $\frac{d\left[ H_{EX} \right]}{dt}={- v}_{max}^{IMP}\frac{\left[ H_{EX} \right]}{K_{M}^{IMP}+\left[ H_{EX} \right]}\left[ cells \right]+k_{cat}^{HrtBA}\left[ HrtBA \right]\frac{\left[ H_{IN} \right]}{K_{M}^{HrtBA}+\left[ H_{IN} \right]}[cells]$ | *(1)* |
| --- | --- |
| $\frac{d\left[ H_{IN} \right]}{dt}=v_{max}^{IMP}\frac{\left[ H_{EX} \right]}{K_{M}^{IMP}+\left[ H_{EX} \right]}-v_{max}^{CON}\frac{\left[ H_{IN} \right]}{K_{M}^{CON}+\left[ H_{IN} \right]}-k_{cat}^{HrtBA}\left[ HrtBA \right]\frac{\left[ H_{IN} \right]}{K_{M}^{HrtBA}+\left[ H_{IN} \right]}- \gamma\left[ H_{IN} \right]$ | *(2)* |
| $\frac{d\left[ cells \right]}{dt}=\beta\frac{\left[ H_{IN} \right]}{K_{M}^{CON}+\left[ H_{IN} \right]}[cells]$ | *(3)* |
| $\frac{d\left[ ChrS \right]}{dt}=\gamma\left[ ChrS_{INI} \right]\left( \frac{1+\omega_{P_{chrSA}}^{ChrA}\left( \frac{\left[ ChrA-P \right]}{\kappa_{P_{chrSA}}^{ChrA}} \right)^{n_{P_{chrsA}}^{ChrA}}}{1+\left( \frac{\left[ ChrA-P \right]}{\kappa_{P_{chrSA}}^{ChrA}} \right)^{n_{P_{chrsA}}^{ChrA}}} \right)- k_{+}^{ChrS}\left[ ChrS \right]\frac{\left[ H_{EX} \right]}{K_{H_{EX}}+\left[ H_{EX} \right]}$ $+k_{1}^{ChrS}\left[ ChrS\sim P \right]\left[ ChrA \right]-\gamma\left[ ChrS \right]$ | *(4)* |
| $\frac{d\left[ ChrS\sim P \right]}{dt}=k_{+}^{ChrS}\left[ ChrS \right]\frac{\left[ H_{EX} \right]}{K_{H_{EX}}+\left[ H_{EX} \right]}-k_{1}^{ChrS}\left[ ChrS\sim P \right]\left[ ChrA \right]-\gamma\left[ ChrS\sim P \right]$ | *(5)* |
| $\frac{d\left[ HrrS \right]}{dt}=\gamma[HrrS_{TOT}]- k_{+}^{HrrS}\left[ HrrS \right]\frac{\left[ H_{EX} \right]}{K_{H_{EX}}+\left[ H_{EX} \right]}+k_{3}^{HrrS}\left[ HrrS\sim P \right]\left[ ChrA \right]-\gamma\left[ HrrS \right]$ | *(6)* |
| $\frac{d\left[ HrrS\sim P \right]}{dt}= k_{+}^{HrrS}\left[ HrrS \right]\frac{\left[ H_{EX} \right]}{K_{H_{EX}}+\left[ H_{EX} \right]}-k_{3}^{HrrS}\left[ HrrS\sim P \right]\left[ ChrA \right]-\gamma\left[ HrrS\sim P \right]$ | *(7)* |
| $\frac{d\left[ ChrA \right]}{dt}=\gamma\left[ ChrA_{INI} \right]\left( \frac{1+\omega_{P_{chrSA}}^{ChrA}\left( \frac{\left[ ChrA-P \right]}{\kappa_{P_{chrSA}}^{ChrA}} \right)^{n_{P_{chrsA}}^{ChrA}}}{1+\left( \frac{\left[ ChrA-P \right]}{\kappa_{P_{chrSA}}^{ChrA}} \right)^{n_{P_{chrsA}}^{ChrA}}} \right)-k_{1}^{ChrS}\left[ ChrS\sim P \right]\left[ ChrA \right]+k_{2}^{ChrS}\left[ ChrS \right]\left[ ChrA\sim P \right]$ $-k_{3}^{HrrS}\left[ HrrS\sim P \right]\left[ ChrA \right]-\gamma\left[ ChrA \right]$ | *(8)* |
| $\frac{d\left[ ChrA\sim P \right]}{dt}=k_{1}^{ChrS}\left[ ChrS\sim P \right]\left[ ChrA \right]-k_{2}^{ChrS}\left[ ChrS \right]\left[ ChrA\sim P \right]+k_{3}^{HrrS}\left[ HrrS\sim P \right]\left[ ChrA \right]-\gamma\left[ ChrA\sim P \right]$ | *(9)* |
| $\frac{d\left[ HrtBA \right]}{dt}=\gamma\left[ HrtBA_{INI} \right]\left( \frac{1+\omega_{P_{hrtBA}}^{ChrA}\left( \frac{\left[ ChrA-P \right]}{\kappa_{P_{hrtBA}}^{ChrA}} \right)^{n_{P_{hrtBA}}^{ChrA}}}{1+\left( \frac{\left[ ChrA-P \right]}{\kappa_{P_{hrtBA}}^{ChrA}} \right)^{n_{P_{hrtBA}}^{ChrA}}} \right)-\gamma\left[ HrtBA \right]$ | *(10)* |
| $\frac{d\left[ ChrS_{TOT}-YFP \right]}{dt}=\gamma\left[ ChrS_{INI} \right]\left( \frac{1+\omega_{P_{chrSA}}^{ChrA}\left( \frac{\left[ ChrA-P \right]}{\kappa_{P_{chrSA}}^{ChrA}} \right)^{n_{P_{chrsA}}^{ChrA}}}{1+\left( \frac{\left[ ChrA-P \right]}{\kappa_{P_{chrSA}}^{ChrA}} \right)^{n_{P_{chrsA}}^{ChrA}}} \right)-\left( \gamma+k_{bl} \right)\left[ ChrS_{TOT}-YFP \right]$ | *(11)* |
| $\frac{d\left[ ChrA_{TOT}-YFP \right]}{dt}=\gamma\left[ ChrA_{INI} \right]\left( \frac{1+\omega_{P_{chrSA}}^{ChrA}\left( \frac{\left[ ChrA-P \right]}{\kappa_{P_{chrSA}}^{ChrA}} \right)^{n_{P_{chrsA}}^{ChrA}}}{1+\left( \frac{\left[ ChrA-P \right]}{\kappa_{P_{chrSA}}^{ChrA}} \right)^{n_{P_{chrsA}}^{ChrA}}} \right)-\left( \gamma+k_{bl} \right)\left[ ChrA_{TOT}-YFP \right]$ | *(12)* |
| $\frac{d\left[ HrtBA-YFP \right]}{dt}=\gamma[Hrt{BA}_{INI}] \left( \frac{1+\omega_{P_{hrtBA}}^{ChrA}\left( \frac{\left[ ChrA-P \right]}{\kappa_{P_{hrtBA}}^{ChrA}} \right)^{n_{P_{hrtBA}}^{ChrA}}}{1+\left( \frac{\left[ ChrA-P \right]}{\kappa_{P_{hrtBA}}^{ChrA}} \right)^{n_{P_{hrtBA}}^{ChrA}}} \right)-(\gamma+k_{bl})\left[ HrtBA-YFP \right]$ | *(13)* |

**Model equations M2:** ODEs of the mathematical model of the *C. glutamicum* heme utilization module

Variables within the model:

| **Name** | **Description** |
| --- | --- |
| *H_EX_* | External heme |
| *H_IN_* | Internal heme |
| *cells* | Cells of *C. glutamicum* |
| *ChrS* | Unphosphorylated histidine kinase ChrS |
| *ChrS~P* | Autophosphorylated histidine kinase ChrS |
| *HrrS* | Unphosphorylated histidine kinase HrrS |
| *HrrS~P* | Autophosphorylated histidine kinase HrrS |
| *HrrA* | Unphosphorylated response regulator HrrA |
| *HrrA~P* | Phosphorylated response regulator HrrA |
| *HmuO* | Heme oxygenase HmuO |
| *DtxR** | Activated form of the iron repressor DtxR |
| *DtxR* | Non-activated form of the iron repressor DtxR |
| *HrrA_TOT_-YFP* | YFP proteins corresponding to the total amount of the response regulator HrrA (The production is under the control of P*_hrrA_* promoter) |
| *HmuO-YFP* | YFP proteins corresponding to the total amount of heme oxygenase HmuO (The production is under the control of P*_hmuO_* promoter) |

ODEs:

| $\frac{d\left[ H_{EX} \right]}{dt}={- v}_{max}^{IMP}\frac{\left[ H_{EX} \right]}{K_{M}^{IMP}+\left[ H_{EX} \right]}\left[ cells \right]$ | *(14)* |
| --- | --- |
| $\frac{d\left[ H_{IN} \right]}{dt}=v_{max}^{IMP}\frac{\left[ H_{EX} \right]}{K_{M}^{IMP}+\left[ H_{EX} \right]}-k_{cat}^{CON}(\left[ E^{CON} \right]+\left[ HmuO \right])\frac{\left[ H_{IN} \right]}{K_{M}^{CON}+\left[ H_{IN} \right]}- \gamma\left[ H_{IN} \right]$ | *(15)* |
| $\frac{d\left[ cells \right]}{dt}=\beta'k_{cat}^{CON}(\left[ E^{CON} \right]+\left[ HmuO \right])\frac{\left[ H_{IN} \right]}{K_{M}^{CON}+\left[ H_{IN} \right]}[cells]$ | *(16)* |
| $\frac{d\left[ ChrS \right]}{dt}=\gamma[ChrS_{TOT}]- k_{+}^{ChrS}\left[ ChrS \right]\frac{\left[ H_{EX} \right]}{K_{H_{EX}}+\left[ H_{EX} \right]}+k_{3}^{ChrS}\left[ ChrS\sim P \right]\left[ HrrA \right]-\gamma\left[ ChrS \right]$ | *(17)* |
| $\frac{d\left[ ChrS\sim P \right]}{dt}=k_{+}^{ChrS}\left[ ChrS \right]\frac{\left[ H_{EX} \right]}{K_{H_{EX}}+\left[ H_{EX} \right]}-k_{3}^{ChrS}\left[ ChrS\sim P \right]\left[ HrrA \right]-\gamma\left[ ChrS\sim P \right]$ | *(18)* |
| $\frac{d\left[ HrrS \right]}{dt}=\gamma[HrrS_{TOT}]- k_{+}^{HrrS}\left[ HrrS \right]\frac{\left[ H_{EX} \right]}{K_{H_{EX}}+\left[ H_{EX} \right]}+k_{1}^{HrrS}\left[ HrrS\sim P \right]\left[ HrrA \right]-\gamma\left[ HrrS \right]$ | *(19)* |
| $\frac{d\left[ HrrS\sim P \right]}{dt}= k_{+}^{HrrS}\left[ HrrS \right]\frac{\left[ H_{EX} \right]}{K_{H_{EX}}+\left[ H_{EX} \right]}-k_{1}^{HrrS}\left[ HrrS\sim P \right]\left[ HrrA \right]-\gamma\left[ HrrS\sim P \right]$ | *(20)* |
| $\frac{d\left[ HrrA \right]}{dt}=\gamma\left[ HrrA_{INI} \right]\left( \frac{1+\omega_{P_{hrrA}}^{HrrA}\left( \frac{\left[ HrrA-P \right]}{\kappa_{P_{hrrA}}^{HrrA}} \right)^{n_{P_{hrrA}}^{HrrA}}}{1+\left( \frac{\left[ HrrA-P \right]}{\kappa_{P_{hrrA}}^{HrrA}} \right)^{n_{P_{hrrA}}^{HrrA}} +\left( \frac{DtxR_{TOT}\frac{\left[ H_{IN} \right]}{K_{H_{IN}}+\left[ H_{IN} \right]}}{\kappa_{P_{hrrA}}^{DtxR}} \right)^{n_{P_{hrrA}}^{DtxR}}} \right)$ $-k_{1}^{HrrS}\left[ HrrS\sim P \right]\left[ HrrA \right]+k_{2}^{HrrS}\left[ HrrS \right]\left[ HrrA\sim P \right]-k_{3}^{ChrS}\left[ ChrS\sim P \right]\left[ HrrA \right]-\gamma\left[ HrrA \right]$ | *(21)* |
| $\frac{d\left[ HrrA\sim P \right]}{dt}=k_{1}^{HrrS}\left[ HrrS\sim P \right]\left[ HrrA \right]-k_{2}^{HrrS}\left[ HrrS \right]\left[ HrrA\sim P \right]+k_{3}^{ChrS}\left[ ChrS\sim P \right]\left[ HrrA \right]-\gamma\left[ HrrA\sim P \right]$ | *(22)* |
| $\frac{d\left[ HmuO \right]}{dt}=\gamma\left[ HmuO_{INI} \right]\left( \frac{1+\omega_{P_{hmuO}}^{HrrA}\left( \frac{\left[ HrrA-P \right]}{\kappa_{P_{hmuO}}^{HrrA}} \right)^{n_{P_{hmuO}}^{HrrA}}}{1+\left( \frac{\left[ HrrA-P \right]}{\kappa_{P_{hmuO}}^{HrrA}} \right)^{n_{P_{hmuO}}^{HrrA}} +\left( \frac{DtxR_{TOT}\frac{\left[ H_{IN} \right]}{K_{H_{IN}}+\left[ H_{IN} \right]}}{\kappa_{P_{hmuO}}^{DtxR}} \right)^{n_{P_{hmuO}}^{DtxR}}} \right)- \gamma[HmuO]$ | *(23)* |
| $\frac{d\left[ HrrA_{TOT}-YFP \right]}{dt}=\gamma\left[ HrrA_{INI} \right]\left( \frac{1+\omega_{P_{hrrA}}^{HrrA}\left( \frac{\left[ HrrA-P \right]}{\kappa_{P_{hrrA}}^{HrrA}} \right)^{n_{P_{hrrA}}^{HrrA}}}{1+\left( \frac{\left[ HrrA-P \right]}{\kappa_{P_{hrrA}}^{HrrA}} \right)^{n_{P_{hrrA}}^{HrrA}} +\left( \frac{DtxR_{TOT}\frac{\left[ H_{IN} \right]}{K_{H_{IN}}+\left[ H_{IN} \right]}}{\kappa_{P_{hrrA}}^{DtxR}} \right)^{n_{P_{hrrA}}^{DtxR}}} \right)$ $-(\gamma+k_{bl})\left[ HrrA_{TOT}-YFP \right]$ | *(24)* |
| $\frac{d\left[ HmuO-YFP \right]}{dt}=\gamma\left[ HmuO_{INI} \right]\left( \frac{1+\omega_{P_{hmuO}}^{HrrA}\left( \frac{\left[ HrrA-P \right]}{\kappa_{P_{hmuO}}^{HrrA}} \right)^{n_{P_{hmuO}}^{HrrA}}}{1+\left( \frac{\left[ HrrA-P \right]}{\kappa_{P_{hmuO}}^{HrrA}} \right)^{n_{P_{hmuO}}^{HrrA}} +\left( \frac{DtxR_{TOT}\frac{\left[ H_{IN} \right]}{K_{H_{IN}}+\left[ H_{IN} \right]}}{\kappa_{P_{hmuO}}^{DtxR}} \right)^{n_{P_{hmuO}}^{DtxR}}} \right)$ $-(\gamma+k_{bl})\left[ HmuO-YFP \right]$ | *(25)* |

DtxR activation:

| $DtxR_{TOT}=DtxR+DtxR^{*}$ | *(26)* |
| --- | --- |
| $DtxR^{*}= DtxR_{TOT}\frac{\left[ H_{IN} \right]}{K_{H_{IN}}+\left[ H_{IN} \right]}$ | *(27)* |
| $DtxR= DtxR_{TOT}\left( 1-\frac{\left[ H_{IN} \right]}{K_{H_{IN}}+\left[ H_{IN} \right]} \right)$ | *(28)* |

**Supplementary Table S1.** Bacterial strains used in this study.

| Strain or plasmid | Relevant characteristics | Source or reference |
| --- | --- | --- |
| *Escherichia coli* | | |
| DH5α | *fhuA2 lac(del)U169 phoA glnV44 Φ80' lacZ(del)M15 gyrA96 recA1 relA1 endA1 thi-1 hsdR17*; for general cloning purposes | Invitrogen |
| BL21(DE3) | B F^–^ *ompT* *gal* *dcm* *lon* *hsdS_B_*(*r_B_*^–^*m_B_*^–^) λ(DE3 [*lacI* *lacUV5*-*T7p07* *ind1* *sam7* *nin5*]) [*malB*^+^]_K-12_(λ^S^); overexpression of proteins. | ([Studier & Moffatt, 1986](#_ENREF_14)) |
| *Corynebacterium glutamicum* | | |
| ATCC 13032 | *C. glutamicum* wild type strain | ([Kinoshita, Udaka, & Shimono, 2004](#_ENREF_10)) |
| ATCC 13032 ∆*hrrS* | Deletion mutant of the open reading frame (orf) encoding the HK HrrS | ([Hentschel et al., 2014](#_ENREF_7)) |
| ATCC 13032 *hrrSQ222A* | Phosphatase=OFF mutant of *hrrS* | ([Hentschel et al., 2014](#_ENREF_7)) |
| ATCC 13032 ∆*chrS* | Deletion mutant of the orf encoding the HK ChrS | ([Hentschel et al., 2014](#_ENREF_7)) |
| ATCC 13032 *chrSQ191A* | Phosphatase=OFF mutant of *hrrS* | ([Hentschel et al., 2014](#_ENREF_7)) |
| ATCC 13032 *chrSH186A* | Kinase=OFF mutant of *chrS* | This study |
| ATCC 13032 ∆*dtxR* | Deletion mutant of the orf encoding DtxR | ([Wennerhold & Bott, 2006](#_ENREF_16)) |
| ATCC 13032 ∆*hrrA* | Deletion mutant of the orf encoding the RR HrrA | ([Frunzke, Gatgens, Brocker, & Bott, 2011](#_ENREF_3)) |

**Supplementary Table S2.** Oligonucleotides used in this study. Restriction sites mutations are underlined.

| **#** | **Name** | **Sequence** | **Special feature** |
| --- | --- | --- | --- |
| 1 | *chrS-fw* | GCGCAAGCTTGTGAAAACTAGCCAAGCGAC | *Hind*III RS |
| 2 | *chrS-rv* | TATACCCGGGTCACTTATCTTGGTCCTTTTG | *Sma*I RS |
| 3 | *chrSH186A* | CCACAGTGTC*AGC*TATTTCGCCCGCTATGCGGGC | *Mutation H186A* |
| 4 | *chrSH186A* | GCCCGCATAGCGGGCGAAATA*GCT*GACACTGTGG | *Mutation H186A* |
| 5 | P*_hmuO_^AAC::TTG^* | CACACCTACATATAGTCCCTTA*CAA*GGAACAATTTTCCGCAACTTTGG | *Mutation P_hmuO_* |
| 6 | P*_hmuO_^AAC::TTG^* | CCAAAGTTGCGGAAAATTGTTCC*TTG*TAAGGGACTATATGTAGGTGTG | *Mutation P_hmuO_* |

**Supplementary Table S3.** Plasmids used in this study. If plasmids were constructed in this study, primers used are indicated in Table S2.

| Reporter plasmids | | | | | |
| --- | --- | --- | --- | --- | --- |
| # | Name | Resistance | Source | | Primer used |
| 1 | pJC1_P*hrtBA-eyfp* | Kanamycin | ([Heyer et al., 2012](#_ENREF_8)) | |  |
| 2 | pJC1_P*hmuO-eyfp* | Kanamycin | ([Heyer et al., 2012](#_ENREF_8)) | |  |
| 3 | pJC1_P*hmuO-eyfp^AAC::TTG^* | Kanamycin | This study | | #5, #6 |
| Plasmids for genomic intergrations | | | | | |
| 4 | pK19_∆*chrS^wt^*::*chrSH184A* | Kanamycin | This study | Cloning: #1, #2  Mut.: #3, #4 | |

**Supplementary Table S4.** Parameters used in the mathematical model of the *C. glutamicum* heme detoxification module.

| Parameter | Notation | Value | Source |
| --- | --- | --- | --- |
| Maximal velocity of heme import via heme transporter HmuTUV | $v_{max}^{IMP}$ | 9.83 x 10^3^ *molecules/min/cell* | Adjusted to match the average growth curve (Fig. 2) |
| Michaelis-Menten constant for heme import via heme transporter HmuTUV | $K_{M}^{IMP}$ | 1.19 x 10^13^ *molecules* |  |
| Maximal velocity of heme consumption via diverse enzymes | $v_{max}^{CON}$ | 7.86 x 10^3^ *molecules/min/cell* |  |
| Michaelis-Menten constant for heme consumption via diverse enzymes | $K_{M}^{CON}$ | 1.84 x 10^6^ *molecules/cell* |  |
| Growth parameter | $\beta$ | 0.04 *1/min* |  |
| Initial OD | $OD_{INI}$ | 2.2 ~ 6.6 x 10^7^ *cells* |  |
| Autophosphorylation threshold of ChrS/HrrS | $K_{H_{EX}}$ | 1.2 x 10^13^ *molecules* | Adjusted to guarantee approximately maximal autophosphorylation rate even for the lowest hemin concentration |
| Autophosphorylation rate of ChrS | $k_{+}^{ChrS}$ | 1 *1/min* | Adjusted to match a rate of autophosphorylation as expected in ([Groban et al., 2009](#_ENREF_6)), taking the ChrS/HrrS levels into account. |
| Autophosphorylation rate of HrrS | $k_{+}^{HrrS}$ | 1 *1/min* |  |
| Effective rate constants of phosphorylation reaction of the cognate kinase ChrS on ChrA | $k_{1}^{ChrS}$ | 3.98 x 10^-3^ *1/molecules/min* | Correspond to the *in vitro* data in ([Hentschel et al., 2014](#_ENREF_7)), multiplied by a factor of 10 due to the observation of ([Gao & Stock, 2017](#_ENREF_5); [Kremling, Kremling, & Bettenbrock, 2009](#_ENREF_11)), that *in vitro* rates are often about 10^1^-10^2^-fold lower than the actual *in vivo* ones. |
| Effective rate constants of dephosphorylation reaction of the cognate kinase ChrS on ChrA | $k_{2}^{ChrS}$ | 3.76 x 10^-2^ *1/molecules/min* |  |
| Effective rate constants of phosphorylation reaction of the non-cognate kinase HrrS on ChrA | $k_{3}^{HrrS}$ | 5.72 x 10^-6^ *1/molecules/min* | Correspond to the *in vitro* data in ([Hentschel et al., 2014](#_ENREF_7)), decreased by a factor of 10, suggested by Fig.S5. |
| Initial ChrS concentration | $ChrS_{INI}$ | 100 *molecules/cell* | Correspond to reference values for total numbers in *E. coli* for several two-component systems ([Cai & Inouye, 2002](#_ENREF_2); [Gao & Stock, 2013](#_ENREF_4); [Li, Burkhardt, Gross, & Weissman, 2014](#_ENREF_12)): Numbers of histidine kinases are within a physiological range of 10^1^-10^3^ and response regulators range between 10^2^ and 10^4^. A 1:1 stoichiometry is assumed for ChrS and ChrA due to the fact that they are within one operon. |
| Initial ChrA concentration | $ChrA_{INI}$ | 100 *molecules/cell* |  |
| Total HrrS concentration | $HrrS_{TOT}$ | 100 *molecules/cell* |  |
| Initial HrtBA concentration | $Hrt{BA}_{INI}$ | 10 *molecules/cell* | Arbitrary choice |
| Turnover rate of heme exporter HrtBA | $k_{cat}^{HrtBA}$ | 20 *molecules/min/transporter* | Adjusted to counteract the import rate under maximal *hrtBA* expression |
| Michaelis-Menten constant for heme export via HrtBA | $K_{M}^{HrtBA}$ | 8 x 10^5^ *molecules/cell* |  |
| Fold-change of P*_chrSA_* promoter | $\omega_{P_{chrSA}}^{ChrA}$ | 70 | Suggested by wildtype data in Fig. S1a; within physiological range of 1-10^4^ (see e.g. ([Lutz & Bujard, 1997](#_ENREF_13))) for promoters with high dynamic range) |
| Fold-change of P*_hrtBA_* promoter | $\omega_{P_{hrtBA}}^{ChrA}$ | 150 |  |
| P*_chrSA_* activation threshold | $\kappa_{P_{chrSA}}^{ChrA}$ | 75 *molecules/cell* | Correspond to the total levels of ChrA~P |
| P*_hrtBA_* activation threshold | $\kappa_{P_{hrtBA}}^{ChrA}$ | 75 *molecules/cell* |  |
| Hill coefficient P*_chrSA_* | $n_{P_{chrSA}}^{ChrA}$ | 1 | Assuming no cooperativity in promoter binding |
| Hill coefficient P*_hrtBA_* | $n_{P_{hrtBA}}^{ChrA}$ | 1 |  |
| Effective rate constant of YFP bleaching and protein degradation | $k_{bl}$ | 0.001 *1/min* | Arbitrary choice |

**Supplementary Table S5.** Additional parameters used in the mathematical model of the *C. glutamicum* heme utilization module (all other parameters are taken from the model of *C. glutamicum* heme detoxification module, cf. Supplementary Table S4).

| Parameter | Notation | Value | Source |
| --- | --- | --- | --- |
| Growth parameter | $\beta'$ | 5 x 10^-6^ *1/molecules* | Adjusted to match the growth rate we observed in the model of *C. glutamicum* heme detoxification module |
| Effective rate constants of phosphorylation reaction of the cognate kinase HrrS on HrrA | $k_{1}^{HrrS}$ | 3.76 x 10^-3^ *1/molecules/min* | Correspond to the *in vitro* data in ([Hentschel et al., 2014](#_ENREF_7)), multiplied by a factor of 10 due to the observation of ([Gao & Stock, 2017](#_ENREF_5); [Kremling et al., 2009](#_ENREF_11)), that *in vitro* rates are often about 10^1^-10^2^-fold lower than the actual *in vivo* ones. |
| Effective rate constants of dephosphorylation reaction of the cognate kinase HrrS on HrrA | $k_{2}^{HrrS}$ | 3.40 x 10^-4^ *1/molecules/min* | Correspond to the *in vitro* data in ([Hentschel et al., 2014](#_ENREF_7)), decreased by a factor of 10, suggested by Fig. S5. |
| Effective rate constants of phosphorylation reaction of the non-cognate kinase ChrS on HrrA | $k_{3}^{ChrS}$ | 1.40 x 10^-3^ *1/molecules/min* | Correspond to the *in vitro* data in ([Hentschel et al., 2014](#_ENREF_7)), multiplied by a factor of 10 due to the observation of  ([Gao & Stock, 2017](#_ENREF_5); [Kremling et al., 2009](#_ENREF_11)), that *in vitro* rates are often about 10^1^-10^2^-fold lower than the actual *in vivo* ones. |
| Total HrrS concentration | $HrrS_{TOT}$ | 100 *molecules/cell* | Reference values for total numbers in *E. coli* for several TCS: Numbers of histidine kinases are within a physiological range of 10^1^-10^3^ and response regulators range between 10^2^ and 10^4^ ([Cai & Inouye, 2002](#_ENREF_2); [Gao & Stock, 2013](#_ENREF_4); [Li et al., 2014](#_ENREF_12)). |
| Initial HrrA concentration | $HrrA_{INI}$ | 100 *molecules/cell* |  |
| Total ChrS concentration | $ChrS_{TOT}$ | 100 *molecules/cell* |  |
| Initial HmuO concentration | $HmuO_{INI}$ | 100 *molecules/cell* | Arbitrary choice |
| Turnover rate of heme consumption via HmuO and diverse other enzymes | $k_{cat}^{CON}$ | 6.04 *molecules/min/transporter* | Adjusted to match the maximal velocity ($v_{max}^{CON}$) and total reaction rate of consumption we observed in the model of *C. glutamicum* heme detoxification module |
| Concentration of other enzymes responsible for heme consumption next to HmuO | $E^{CON}$ | 1000 *molecules//cell* |  |
| Michaelis-Menten constant for heme consumption via HmuO and diverse other enzymes | $K_{M}^{CON}$ | 1.84 x 10^6^ *molecules/cell* |  |
| Total DtxR concentration | $DtxR_{TOT}$ | 1000 *molecules/cell* | Arbitrary choice |
| Activation threshold of DtxR | $K_{H_{IN}}$ | 8 x 10^5^ *molecules/cell* | Adjusted to guarantee a sufficient activation of iron repressor DtxR for the highest hemin concentration. |
| Fold-change of P*_hrrA_* promoter | $\omega_{P_{hrrA}}^{HrrA}$ | 5 | Suggested by wildtype data in Fig. S1a; within physiological range of 1-10^4^ (see e.g. ([Lutz & Bujard, 1997](#_ENREF_13)) for promoters with high dynamic range) |
| Fold-change of P*_hmuO_* promoter | $\omega_{P_{hmuO}}^{ChrA}$ | 50 |  |
| P*_hrrA_* activation threshold | $\kappa_{P_{hrrA}}^{HrrA}$ | 100 *molecules/cell* | Correspond to the total levels of ChrA~P and activated DtxR. Experimental Data suggest a significant higher activation via HrrA than the repression via DtxR on P*_hrrA_* |
| P*_hmuO_* activation threshold | $\kappa_{P_{hmuO}}^{HrrA}$ | 300 *molecules/cell* |  |
| P*_hmuO_* threshold of DtxR repression | $\kappa_{P_{hmuO}}^{DtxR}$ | 300 *molecules/cell* |  |
| P*_hrrA_* threshold of DtxR repression | $\kappa_{P_{hrrA}}^{DtxR}$ | 500 *molecules/cell* |  |
| Hill coefficient of HrrA binding to P*_hrrA_* | $n_{P_{hrrA}}^{HrrA}$ | 1 | Assuming no cooperativity in promoter binding on P*_hrrA_* |
| Hill coefficient of HrrA binding to P*_hmuO_* | $n_{P_{hmuO}}^{HrrA}$ | 1 |  |
| Hill coefficient of DtxR binding to P*_hmuO_* | $n_{P_{hmuO}}^{DtxR}$ | 7 | Adjusted to have a strong repressive effect on P*_hmuO_*/P*_hrtBA_* |
| Hill coefficient of DtxR  binding to P*_hmrrA_* | $n_{P_{hrrA}}^{DtxR}$ | 7 |  |

**Supplementary Table S6**. Transformation of the units within the mathematical models

| Experimental unit | Unit within the mathematical models | Source |
| --- | --- | --- |
| OD = 1 | ~ 3 x 10^8^ *cells/mL* | ([Unthan et al., 2015](#_ENREF_15)) |
| Heme [*μM]* in the growth medium | ~ 6 x 10^13^ *molecules* heme in the growth medium | Assuming a reaction volume of ~100 *μL* |
| Free heme [*μM]* in the cell | ~1000 heme *molecules/cell* | Corresponds to a cell size of ~ 1*μm^3^* [REF] |

S****upporting References****

Bintu, L., Buchler, N. E., Garcia, H. G., Gerland, U., Hwa, T., Kondev, J., & Phillips, R. (2005). Transcriptional regulation by the numbers: models. *Curr Opin Genet Dev, 15*(2), 116-124. doi: 10.1016/j.gde.2005.02.007

Cai, S. J., & Inouye, M. (2002). EnvZ-OmpR interaction and osmoregulation in Escherichia coli. *J Biol Chem, 277*(27), 24155-24161. doi: 10.1074/jbc.M110715200

Frunzke, J., Gatgens, C., Brocker, M., & Bott, M. (2011). Control of heme homeostasis in *Corynebacterium glutamicum* by the two-component system HrrSA. *J Bacteriol, 193*(5), 1212-1221. doi: 10.1128/jb.01130-10

Gao, R., & Stock, A. M. (2013). Evolutionary tuning of protein expression levels of a positively autoregulated two-component system. *PLoS Genet, 9*(10), e1003927. doi: 10.1371/journal.pgen.1003927

Gao, R., & Stock, A. M. (2017). Quantitative Kinetic Analyses of Shutting Off a Two-Component System. *MBio, 8*(3). doi: 10.1128/mBio.00412-17

Groban, E. S., Clarke, E. J., Salis, H. M., Miller, S. M., & Voigt, C. A. (2009). Kinetic buffering of cross talk between bacterial two-component sensors. *J Mol Biol, 390*(3), 380-393. doi: 10.1016/j.jmb.2009.05.007

Hentschel, E., Mack, C., Gatgens, C., Bott, M., Brocker, M., & Frunzke, J. (2014). Phosphatase activity of the histidine kinases ensures pathway specificity of the ChrSA and HrrSA two-component systems in *Corynebacterium glutamicum*. *Mol Microbiol, 92*(6), 1326-1342. doi: 10.1111/mmi.12633

Heyer, A., Gatgens, C., Hentschel, E., Kalinowski, J., Bott, M., & Frunzke, J. (2012). The two-component system ChrSA is crucial for haem tolerance and interferes with HrrSA in haem-dependent gene regulation in *Corynebacterium glutamicum*. *Microbiology, 158*(Pt 12), 3020-3031. doi: 10.1099/mic.0.062638-0

Keppel, M., Davoudi, E., Gätgens, C., & Frunzke, J. (2018). Membrane Topology and Heme Binding of the Histidine Kinases HrrS and ChrS in *Corynebacterium glutamicum*. *Front Microbiol, 9*(183). doi: 10.3389/fmicb.2018.00183

Kinoshita, S., Udaka, S., & Shimono, M. (2004). Studies on the amino acid fermentation. Part 1. Production of L-glutamic acid by various microorganisms. *J Gen Appl Microbiol, 50*(6), 331-343.

Kremling, A., Kremling, S., & Bettenbrock, K. (2009). Catabolite repression in *Escherichia coli*- a comparison of modelling approaches. *FEBS J, 276*(2), 594-602. doi: 10.1111/j.1742-4658.2008.06810.x

Li, G. W., Burkhardt, D., Gross, C., & Weissman, J. S. (2014). Quantifying absolute protein synthesis rates reveals principles underlying allocation of cellular resources. *Cell, 157*(3), 624-635. doi: 10.1016/j.cell.2014.02.033

Lutz, R., & Bujard, H. (1997). Independent and tight regulation of transcriptional units in *Escherichia coli* via the LacR/O, the TetR/O and AraC/I1-I2 regulatory elements. *Nucleic Acids Res, 25*(6), 1203-1210.

Studier, F. W., & Moffatt, B. A. (1986). Use of bacteriophage T7 RNA polymerase to direct selective high-level expression of cloned genes. *J Mol Biol, 189*(1), 113-130.

Unthan, S., Baumgart, M., Radek, A., Herbst, M., Siebert, D., Bruhl, N., . . . Noack, S. (2015). Chassis organism from Corynebacterium glutamicum--a top-down approach to identify and delete irrelevant gene clusters. *Biotechnol J, 10*(2), 290-301. doi: 10.1002/biot.201400041

Wennerhold, J., & Bott, M. (2006). The DtxR regulon of *Corynebacterium glutamicum*. *J Bacteriol, 188*(8), 2907-2918. doi: 10.1128/jb.188.8.2907-2918.2006
